# PCBP2 maintains antiviral signaling homeostasis by regulating cGAS enzymatic activity via antagonizing its condensation

**DOI:** 10.1038/s41467-022-29266-9

**Published:** 2022-03-23

**Authors:** Haiyan Gu, Jing Yang, Jiayu Zhang, Ying Song, Yao Zhang, Pengfei Xu, Yuanxiang Zhu, Liangliang Wang, Pengfei Zhang, Lin Li, Dahua Chen, Qinmiao Sun

**Affiliations:** 1 State Key Laboratory of Membrane Biology, Institute of Zoology, Chinese Academy of Sciences, Datun Road, Chaoyang District, Beijing, 100101 China; 2 Institute of Biomedical Research, Yunnan University, Kunming, 650500 China; 3 Institute of Stem Cells and Regeneration, Chinese Academy of Sciences, Beijing, 100101 China; 4 School of Life Sciences, University of Chinese Academy of Sciences, Beijing, 100049 China

**Keywords:** Signal transduction, Innate immunity, Cell signalling, Proteomics

## Abstract

Cyclic GMP-AMP synthase (cGAS) plays a major role in detecting pathogenic DNA. It produces cyclic dinucleotide cGAMP, which subsequently binds to the adaptor protein STING and further triggers antiviral innate immune responses. However, the molecular mechanisms regulating cGAS enzyme activity remain largely unknown. Here, we characterize the cGAS-interacting protein Poly(rC)-binding protein 2 (PCBP2), which plays an important role in controlling cGAS enzyme activity, thereby mediating appropriate cGAS-STING signaling transduction. We find that PCBP2 overexpression reduces cGAS-STING antiviral signaling, whereas loss of PCBP2 significantly increases cGAS activity. Mechanistically, we show that PCBP2 negatively regulates anti-DNA viral signaling by specifically interacting with cGAS but not other components. Moreover, PCBP2 decreases cGAS enzyme activity by antagonizing cGAS condensation, thus ensuring the appropriate production of cGAMP and balancing cGAS-STING signal transduction. Collectively, our findings provide insight into how the cGAS-mediated antiviral signaling is regulated.

## Abstract (web summary)

cGAS senses viral DNA and forms cytosolic cGAS-DNA granules to mediate anti-DNA viral signaling pathway. Here the authors show that PCBP2 interacts with cGAS and antagonizes condensation of cGAS-DNA granules, thus maintaining host immune homeostasis.

## Introduction

The innate immune system serves as the first line of host defense against viral infection using a number of sensors known as pattern recognition receptors (PRRs). PRRs specifically recognize conserved microbial components termed pathogen-associated molecular patterns (PAMPs). Nucleic acids derived from viruses function as PAMPs and are detected by PRRs, consequently initiating the innate immune responses and resulting in the production of type I interferons (IFNs) and proinflammatory cytokines^1–3^.

Cyclic GMP-AMP synthase (cGAS) functions as a cytosolic DNA sensor and plays a critical role in the innate immune response against DNA virus infection. cGAS catalyzes the conversion of ATP and GTP to cGAMP. cGAMP functions as an endogenous second messenger by binding to the endoplasmic reticulum membrane protein STING and activating the transcription factors IRF3 and NF-κB through the kinases TBK1/IKKi and IKK complex, respectively, subsequently inducing the production of IFNs and proinflammatory cytokines^4,5^. cGAS remains in an inactive state in resting cells and is activated upon binding to DNA^6^. Because cGAS can detect both self and non-self-DNA, its activity must be strictly regulated to maintain the balance in innate immune responses, subsequently preventing the development of autoimmune diseases^7^. cGAS activity is regulated by multiple factors, including protein post-translational modifications, such as phosphorylation^8,9^, glutamylation^10^, sumoylation^11,12^, acetylation^13,14^, and ubiquitination^15,16^. In addition to these post-translational modifications, cGAS function has been found to be negatively regulated by several other factors. For example, previous studies demonstrated that Caspase 1/3 cleaves cGAS to inhibit cGAS-STING signaling^17,18^, and Gasdermin D suppresses cGAS activity by disrupting K + efflux^19^. The autophagy protein Beclin-1 inhibits cGAS enzymatic activity^20^, and p62 regulates the autophagic degradation of cGAS^15,21^. Although previous studies have made significant progress in understanding the mechanisms regulating cGAS activity, how cGAS activity is tightly controlled to maintain immune homeostasis remains unclear.

Previous studies have indicated that cGAS can form dimers, undergo aggregation after sensing cytosolic DNA^6,22,23^, and form liquid droplets in vitro and in vivo^24^. DNA-induced liquid phase condensation of cGAS promotes its enzymatic activity to increase cGAMP production and plays an important role in antiviral signaling^24^. However, the mechanisms that dynamically regulate cGAS condensation to initiate an appropriate immune response to pathogens and prevent overreaction are poorly understood.

Poly(rC)-binding protein 2 (PCBP2) belongs to a class of proteins that bind to poly(C) stretches of both RNA and DNA^25–27^, and it plays important roles in regulating mRNA stability^28,29^, protein translation^30,31^, and protein–protein interactions^32,33^. A previous study demonstrated that PCBP2 is a negative modulator of innate immune responses against RNA virus infection by regulating MAVS stability via the HECT ubiquitin ligase AIP4^32^. However, whether PCBP2 is involved in cGAS-STING signaling is still unclear.

To further explore the mechanism underlying the regulation of cGAS in the context of viral infection, we performed co-immunoprecipitation (Co-IP) experiments in combination with mass spectrometry assays and identified PCBP2 as a cGAS-interacting protein. We showed that PCBP2 overexpression significantly impaired cGAS-STING signaling, whereas PCBP2 deficiency remarkably enhanced the innate immune response induced by DNA stimulation or DNA virus infection in various cell lines. In addition, PCBP2 reduction in mouse embryonic fibroblasts (MEFs) apparently reduced HSV-1 virus replication. Mechanistically, we found that PCBP2 could interact with cGAS and negatively affect its enzymatic activity. Furthermore, we determined that PCBP2 evidently attenuated cGAS aggregates and cGAS-DNA phase separation. Collectively, we revealed that PCBP2 negatively regulates cGAS-mediated innate immune responses against DNA virus infection by attenuating the enzyme activity of cGAS. Thus, our findings provide insight into the mechanisms of innate immune responses against DNA virus infection that function to maintain immune homeostasis.

## Results

### Identification of PCBP2 as a cGAS-interacting factor

**Fig. 1:**
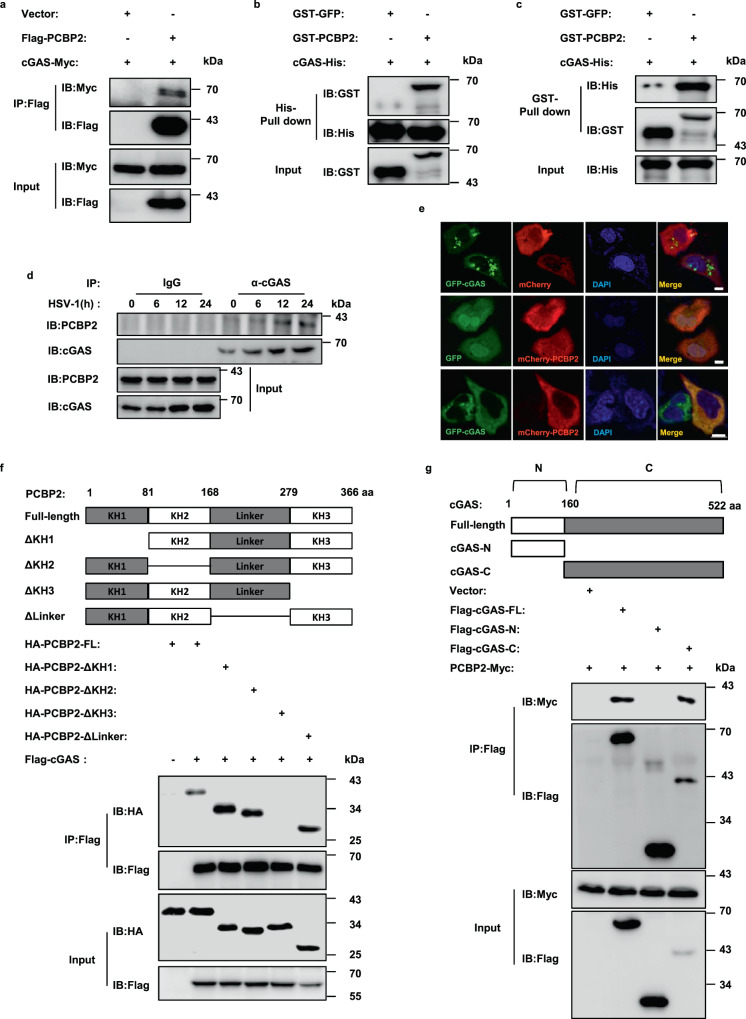
Identification of PCBP2 as a cGAS-interacting factor. **a** HEK293T cells were co-transfected with Myc-tagged cGAS and Flag-tagged PCBP2 or an empty vector, and cell lysates were immunoprecipitated with anti-Flag beads, followed by immunoblot analysis. **b**, **c** Purified cGAS-His was incubated with GST-PCBP2 or GST-GFP and then pulled down with Ni-Sepharose beads (**b**) or glutathione-Sepharose beads (**c**). Western blotting was performed to detect the presence of His-tagged cGAS and GST-tagged PCBP2 proteins. **d** THP-1 cells were mock-infected or infected with HSV-1 for the indicated times, and then cell lysates were immunoprecipitated with a rabbit anti-cGAS antibody or control IgG, followed by immunoblotting. **e** HeLa cells were transfected with the indicated expression plasmids. Twenty-four hours after transfection, the cells were fixed, stained with DAPI, and observed by confocal microscopy. Scale bars, 10 μm. **f** Schematic diagram of PCBP2 full-length (FL) and its truncated mutants (upper panels). HEK293T cells were transfected with the indicated plasmids, and cell lysates were immunoprecipitated with anti-Flag beads, followed by immunoblotting (lower panels). **g** Schematic diagram of cGAS and its truncated mutants (upper panels). HEK293T cells were co-transfected PCBP2 with cGAS, its truncated mutants, or an empty vector, as indicated. The cell lysates were immunoprecipitated with anti-Flag beads, followed by immunoblotting with the indicated antibodies (lower panels). Source data are provided as a Source data file.

To better understand the molecular basis underlying the regulation of cGAS upon DNA virus infection, we aimed to identify proteins associated with cGAS. We infected human HEK293A cells with lentivirus expressing SFB-tagged cGAS or an empty vector for 48 h and then infected with (or without) herpes simplex virus type 1 (HSV-1), a double-stranded DNA virus. The cell lysis was collected to perform Co-IP experiments, followed by mass spectrometry analysis. Using this approach, we identified a number of proteins that were potentially associated with cGAS (Fig. S1a, b). Among these candidates, PCBP2 was of particular interest because it has been shown to regulate the MAVS-mediated antiviral response against RNA virus infection^32,33^. To verify the association of PCBP2 with cGAS, we co-expressed PCBP2 and cGAS in HEK293T cells and performed additional Co-IP experiments. The results showed that PCBP2 was associated with cGAS (Fig. 1a). To determine whether the interaction between PCBP2 and cGAS is direct, we purified recombinant cGAS-His and GST-PCBP2 from *Escherichia coli* (*E. coli*) and performed in vitro pull-down experiments. As shown in Fig. 1b, c, cGAS-His efficiently pulled down GST-PCBP2 but not the GST-GFP control, and GST-PCBP2 but not the GST-GFP control pulled down cGAS-His, suggesting that PCBP2 directly binds to cGAS. Moreover, we performed direct binding surface plasmon resonance (SPR) assays to examine the kinetics of purified recombinant cGAS binding to PCBP2 and obtained the consistent results of Co-IP between cGAS and PCBP2 (Fig. S2a). Next, to determine whether endogenous cGAS forms a complex with PCBP2 under physiological or HSV-1 infection conditions, we used anti-cGAS antibody to perform Co-IP assays in THP-1 cells. As shown in Fig. 1d, the association between PCBP2 and cGAS was detected under both normal physiological and viral infection conditions. Interestingly, we found that HSV-1 infection enhanced the signal of the cGAS-PCBP2 interaction in THP-1 cells. Because cGAS protein levels were increased upon HSV-1 infection (Fig. 1d), we sought to determine whether the increased cGAS-PCBP2 interaction induced by HSV-1 infection is due to increased cGAS expression, or HSV-1 infection. To do so, we employed STING knockout THP1 cells, in which the endogenous cGAS protein was not enhanced by HSV-1 infection. As shown by Co-IP analysis, HSV-1 infection enhanced cGAS-PCBP2 interaction (Fig. S2b). In addition, immunostaining assay results showed that PCBP2 was co-localized with cGAS following their co-transfection in HeLa cells (Figs. 1e and S2c). Similar co-localization patterns were observed when endogenous cGAS and PCBP2 were analyzed in THP-1 cells with and without HSV-1 infection (Fig. S2d, e). To strengthen our findings, we employed the split GFP system, which was widely used to determine the protein–protein interaction in cells, to trace the behavior of cGAS and PCBP2 in vivo. As shown in Fig. S2f, g, the PCBP2-cGAS interaction occurred in HeLa cells and HSV-1 infection appeared to increase the cGAS-PCBP2 interaction. Collectively, these findings suggest that PCBP2 and cGAS form a complex in cells and the association of two proteins is likely modulated by viral infection.

PCBP2 contains three heterogeneous nuclear ribonucleoprotein K-homology (KH) domains and a linker region^32^. To map which domain is required for PCBP2 to associate with cGAS, we generated a series of deleted forms of PCBP2, in which the KH1, KH2, linker, or KH3 domain was deleted. As shown by the results of Co-IP assays, KH3 but not KH1, KH2, or the linker was important for PCBP2-cGAS interactions (Fig. 1f). Moreover, SPR assays consistently showed that PCBP2 KH3-deleted mutant (PCBP2-ΔKH3) failed to interact with cGAS (Fig. S2a). Next, we aimed to identify the domain in cGAS that is required for its interaction with PCBP2. Because cGAS contains a less-conserved N-terminal disordered region and highly conserved C-terminal catalytic domains harboring NTase and Mab21 domains^4^, we generated two deleted forms of cGAS for subsequent Co-IP experiments. As shown in Fig. 1g, C-terminal domain but not the N-terminal domain, were essential for the association between cGAS and PCBP2. These data suggest that the interaction between PCBP2 and cGAS occurs in a domain-dependent manner.

### PCBP2 decreases cGAS-STING antiviral signaling

**Fig. 2:**
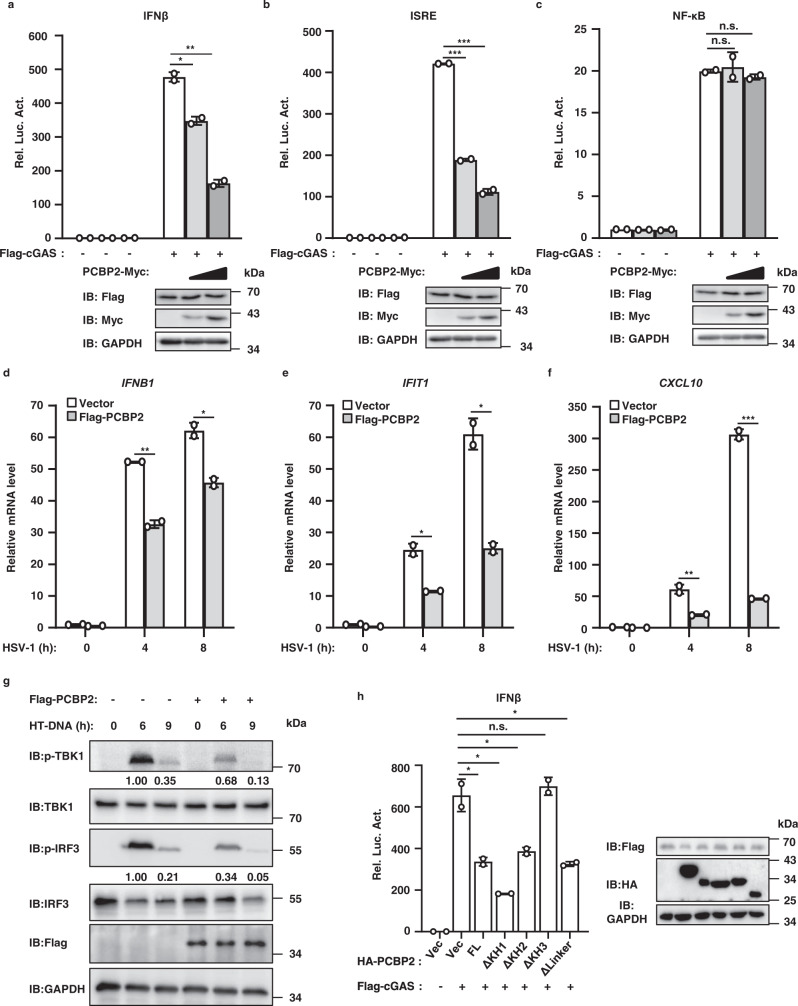
Overexpression of PCBP2 antagonizes cGAS-STING-mediated antiviral signaling. **a**–**c** HEK293T cells stably expressing Flag-STING were transfected with the indicated expression plasmids together with luciferase reporter constructs driven by the promoter of genes encoding IFN-β (**a**), ISRE (**b**), or NF-ĸB (**c**). Renilla was used as an internal control. Twenty-four hours after transfection, cells were lysed for luciferase reporter assays (upper panel) and immunoblotting (lower panels). **a** *p* = 0.01; *p* = 0.0016. **b** *p* < 0.0001; *p* = 0.0003 in sequence. **d**–**f** THP-1 cells were infected with a lentivirus expressing PCBP2 or an empty vector for 48 h, followed by HSV-1 infection (MOI = 5) for  the indicated times. The cells were harvested to measure the transcriptional levels of *IFNB1* (**d**), *IFIT1* (**e**), and *CXCL10* (**f**) by qRT-PCR analysis. **d** *p* = 0.0019 (4 h); *p* = 0.0148 (8 h). **e** *p* = 0.0108 (4 h); *p* = 0.0103 (8 h). **f** *p* = 0.0096 (4 h); *p* = 0.0008 (8 h). **g** THP-1 cells were infected with a lentivirus expressing PCBP2 or an empty vector for 48 h, followed by HT-DNA transfection for the indicated times. Cell lysates were resolved by SDS-PAGE, followed by immunoblotting. **h** HEK293T cells stably expressing Flag-STING were co-transfected with cGAS and PCBP2 full-length (FL) or its mutant constructs together with the IFNβ-Luc reporter, followed by luciferase reporter assays (left panel) and immunoblotting (right panels). *p* = 0.0305 (FL); *p* = 0.0134 (ΔKH1); *p* = 0.0417 (ΔKH2); *p* = 0.5593 (ΔKH3); *p* = 0.0274 (ΔLinker). The data shown in **a**–**f**, **h** are from one representative experiment of at least 3 biological independent experiments (mean ± SD, *n* = 2 independent samples). The two-tailed Student’s *t*-test was used to analyze statistical significance. *p < 0.05; **p < 0.01; ***p < 0.001; n.s. not significant versus the control groups. Source data are provided as a Source data file.

Given that PCBP2 associates with cGAS and that this association is regulated by viral infection, we reasoned that PCBP2 might regulate cGAS-STING-mediated antiviral signaling by targeting cGAS. We first generated a cell-based luciferase reporter system to test the role of PCBP2 in the type-I IFN signaling induced by cGAS. In the control experiments, the co-expression of cGAS and STING activated the IFNβ promoter in HEK293T cells. In contrast, we found that PCBP2 overexpression significantly downregulated the activity of IFNβ induced by the co-expression of cGAS and STING, suggesting a role of PCBP2 in balancing cGAS-STING signaling activity (Fig. 2a). Of note, the stimulation of IFN-β requires the coordinated activation of both IRF3 and NF-κB transcription factors. To understand how PCBP2 regulates cGAS-STING signaling, we used an IFN-stimulated response element (ISRE) luciferase reporter that can be activated by IRF3 and a NF-κB luciferase reporter to perform cell-based luciferase assays. As shown in Fig. 2b, c, PCBP2 overexpression significantly reduced the activation of the ISRE reporter that was stimulated by the co-expression of cGAS and STING but had no effect on the NF-κB reporter. These results suggest that PCBP2 attenuated the cGAS-mediated induction of IFNβ activation by regulating IRF3 activation.

To verify our observation from reporter assays, we then performed quantitative reverse transcription PCR (qRT-PCR) analysis to measure the transcriptional levels of antiviral genes, such as *IFNB1*, *IFIT1*, and *CXCL10*, in human macrophage THP-1 cells with or without PCBP2 overexpression following the infection of HSV-1. As shown in Fig. 2d–f, PCBP2 overexpression significantly reduced the mRNA levels of *IFNB1, IFIT1*, and *CXCL10* induced by HSV-1 infection. Given that the phosphorylation of TBK1 and IRF3 is a hallmark of activated antiviral signaling, we then tested whether PCBP2 overexpression affects the phosphorylation of these factors. Western blotting results showed that PCBP2 overexpression in THP-1 cells significantly reduced the levels of phosphorylated TBK1 and IRF3 induced by transfection with Herring testis DNA (HT-DNA), which mimics viral DNA ligands that bind to cGAS (Fig. 2g), or HSV-1 infection (Fig. S3a). Consistent with reporter assays, PCBP2 overexpression had no effects on the levels of phosphorylated P65 induced by HSV-1 infection (Fig. S3a). In addition, we tested whether PCBP2 regulates cGAS signaling in a mouse cell line and found that overexpressed PCBP2 significantly reduced HT-DNA-induced production of *Ifnb1* and *Ifit1* but not *Il-6* in mouse fibroblast L929 cells (Fig. S3b–d). Collectively, these findings suggest that PCBP2 overexpression antagonizes cGAS-mediated antiviral signaling.

The experiments described above indicated that the KH3 domain of PCBP2 is critical for its association with cGAS. We then examined whether the deletion of this domain affects the ability of PCBP2 to regulate cGAS signaling. As shown in Fig. 2h, luciferase reporter assays revealed that deletion of the KH3 domain of PCBP2 resulted in the loss of its ability to reduce cGAS-STING activation, whereas other deletion mutants exhibited a similar reduced ability as full-length PCBP2. These results suggest that the KH3-mediated PCBP2-cGAS interaction is important for PCBP2 to antagonize cGAS-mediated antiviral signaling.

### Depletion of PCBP2 enhances cGAS-STING signaling

**Fig. 3:**
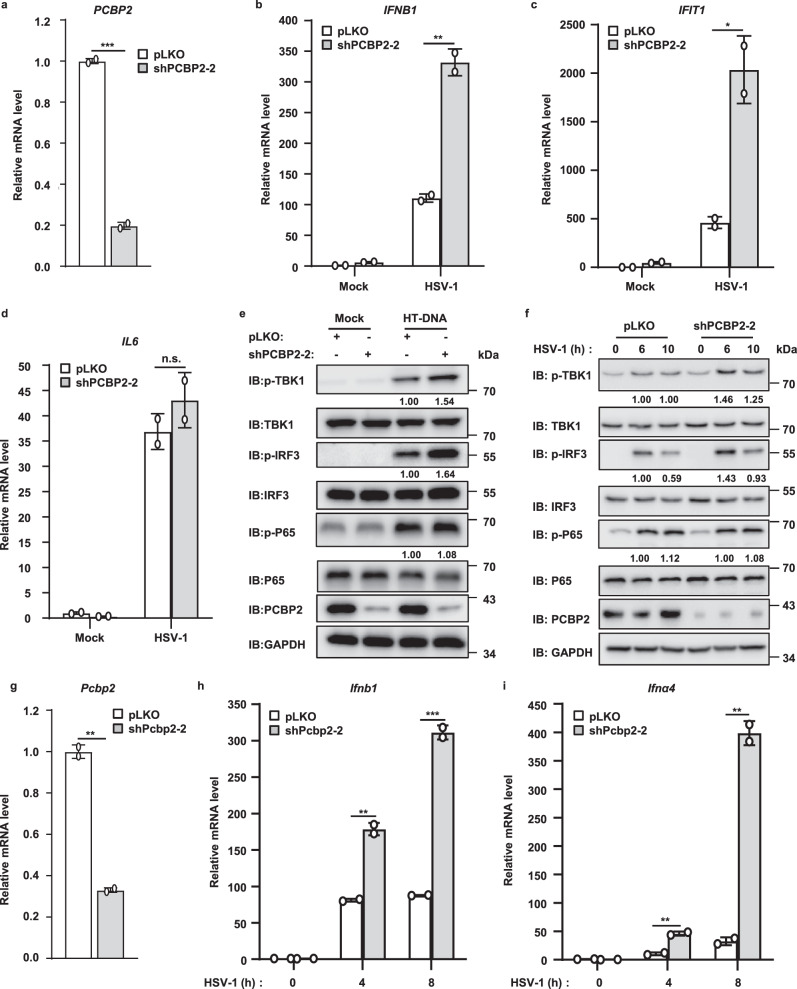
Knockdown of *PCBP2* increases cGAS-STING signaling. **a**–**d** THP-1 cells were infected with lentiviruses-based shRNA targeting *PCBP2* (shPCBP2-2) or an empty vector for 48 h and then left uninfected or infected with HSV-1 (MOI = 5) for 6 h. The cells were harvested for qRT-PCR assays to measure the transcriptional levels of *PCBP2* (**a**), *IFNB1* (**b**), *IFIT1* (**c**), and *IL6* (**d**). **a** *p* = 0.0004. **b** *p* = 0.0053. **c** *p* = 0.0243. **e** THP-1 cells were infected with a lentivirus-based shRNA targeting *PCBP2* or an empty vector for 48 h, and then transfected with HT-DNA (2 μg/ml) for 6 h or mock-treated (Mock). The cells were lysed and followed by immunoblotting. **f** THP-1 cells were infected with a lentivirus-based shRNA targeting PCBP2 or an empty vector for 48 h, and then infected with HSV-1 (MOI = 10) for the indicated times. The cells were lysed and followed by immunoblotting. **g**–**i** RAW264.7 cells were infected with a lentivirus-based shRNA targeting *Pcbp2* (sh*Pcbp2*-2) or an empty vector for 48 h and then infected with HSV-1 (MOI = 5) for the indicated times. Transcriptional levels of *Pcbp2* (**g**), *Ifnb1* (**h**), and *Ifnα4* (**i**) were measured by qRT-PCR assays. **g** *p* = 0.0013. **h** *p* = 0.004 (4 h); *p* = 0.0009 (8 h). **i** *p* = 0.0069 (4 h); *p* = 0.0018 (8 h). The data shown in **a**–**d**, **g**–**i** are from one representative experiment of at least 3 biological independent experiments (mean ± SD, *n* = 2 independent samples). The two-tailed Student’s *t*-test was used to analyze statistical significance. *p < 0.05; **p < 0.01; ***p < 0.001; n.s. not significant versus the control groups. Source data are provided as a Source data file.

Next, we tested whether the knockdown of endogenous PCBP2 affects cGAS-mediated antiviral signaling. For this purpose, we generated two lentiviral constructs expressing short hairpin RNAs (shRNAs) against different regions of human *PCBP2* (shPCBP2-1 and shPCBP2-2). We infected THP-1 cells with a lentivirus carrying shPCBP2-1 or shPCBP2-2 and then performed western blotting. The results showed that shPCBP2-2 displayed a higher PCBP2 knockdown efficiency (Fig. S4a), and thus it was selected for further experiments. As shown in Fig. 3a–c, PCBP2 knockdown significantly enhanced the mRNA level of *IFNB1* and *IFIT1* induced by HSV-1 infection, whereas the mRNA expression of *IL6*, a downstream target of NF-κB, was not significantly affected by PCBP2 knockdown compared with control cells (Fig. 3d). Consistently, the knockdown of PCBP2 in THP-1 cells further elevated the levels of phosphorylated TBK1 and IRF3, but not p65, stimulated by HT-DNA and HSV-1 infection (Fig. 3e, f). To obtain more evidence supporting our conclusion, we studied the function of PCBP2 in mouse cells. We infected mouse macrophage Raw 264.7 cells with two shRNAs specifically targeting the coding region of mouse *Pcbp2*. As shown by qRT-PCR analysis, shPcbp2-2 exhibited a higher knockdown efficiency, and shPcbp2-2-mediated Pcbp2 knockdown significantly increased the production of *Ifnb1, Ifnα4*, and *Cxcl10* mRNA following HSV-1 virus infection compared with control cells (Fig. S4b–e). We also observed similarly enhanced antiviral responses at different time points after HSV-1 infection (Fig. 3g–i). Collectively, these findings further support the notion that PCBP2 acts as a negative regulator to balance cGAS signaling.

### Enhanced cGAS-STING signaling in *Pcbp2*-deficient cells

**Fig. 4:**
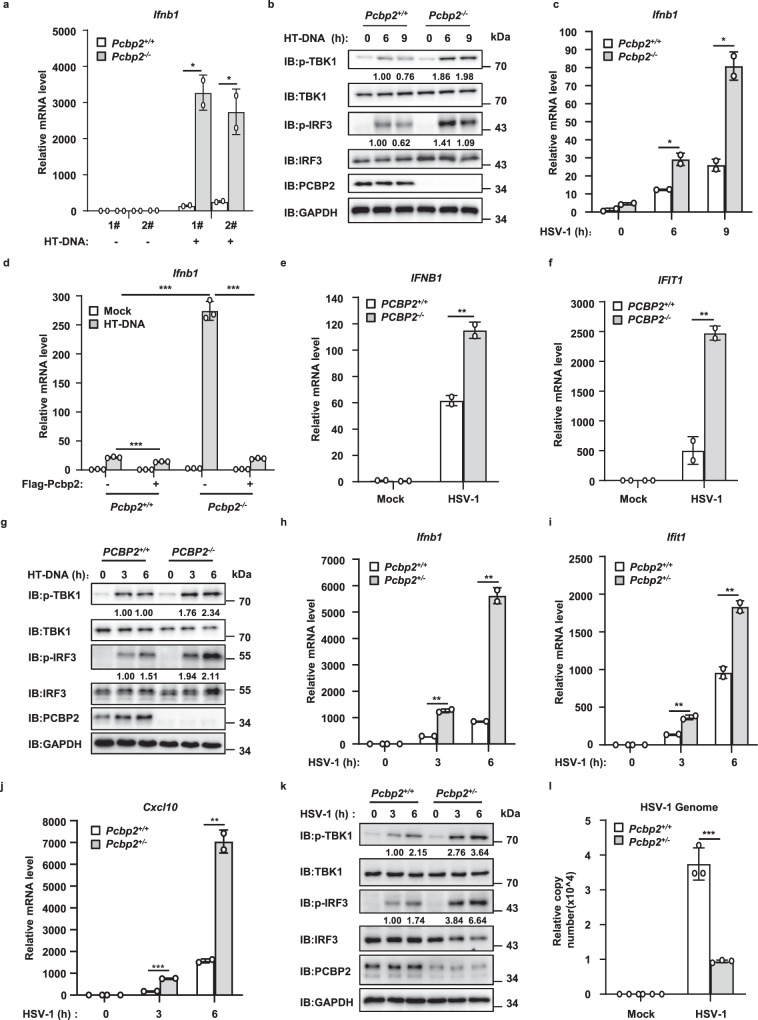
PCBP2 deficiency augments cGAS-STING signaling. **a** Two different clones from wild-type and *Pcbp2*-deficient L929 cells were transfected with HT-DNA (2 μg/ml) for 6 h, followed by qRT-PCR analysis. *p* = 0.0119 (1#); *p* = 0.0306 (2#). **b** *Pcbp2*^*+/+*^ and *Pcbp2*^*−/−*^ L929 cells were transfected with HT-DNA (2 μg/ml) for the indicated times, followed by immunoblotting. **c** *Pcbp2*^*+/+*^ and *Pcbp2*^*−/−*^ L929 cells were infected with HSV-1 (MOI = 5) for the indicated times, followed by qRT-PCR analysis. *p* = 0.0202 (6 h); *p* = 0.0117 (9 h). **d** *Pcbp2*^*+/+*^ and *Pcbp2*^*−/−*^ L929 cells were first infected with a lentivirus expressing PCBP2 or an empty vector. After 48 h of infection, cells were then transfected with or without HT-DNA (2 μg/ml) for 6 h, followed by qRT-PCR analysis. *p* = 0.0007; *p* < 0.0001; *p* < 0.0001 in sequence. **e**–**f** *PCBP2*^*+/+*^ and *PCBP2*^*−/−*^ THP-1 cells were infected with HSV-1 (MOI = 5) for 6 h, followed by qRT-PCR analysis to measure the mRNA levels of *IFNB1* (**e**) and *IFIT1* (**f**). **e** *p* = 0.0092. **f** *p* = 0.0087. **g** *PCBP2*^*+/+*^ and *PCBP2*^*−/−*^ THP-1 cells were transfected with HT-DNA (2 μg/ml) for the indicated times, followed by immunoblotting. **h**–**j** *Pcbp2*^*+/+*^ and *Pcbp2*^*+/−*^ MEFs were infected with HSV-1 (MOI = 5) for the indicated times and then lysed for the quantification of *Ifnb1* (**h**), *Ifit1* (**i**), and *Cxcl10* (**j**) mRNA levels by qRT-PCR. **h** *p* = 0.0027 (3 h); *p* = 0.002 (6 h). **i** *p* = 0.0097 (3 h); *p* = 0.0077 (6 h); **j** *p* = 0.0004 (3 h); *p* = 0.0047 (6 h). **k** *Pcbp2*^*+/+*^ and *Pcbp2*^*+/−*^ MEFs were infected with HSV-1 (MOI = 5) for the indicated times and then lysed for immunoblotting. **l** *Pcbp2*^*+/+*^ and *Pcbp2*^*+/−*^ MEFs were infected with HSV-1 (MOI = 0.1) for 18 h. The genomic DNA was extracted, and the relative HSV-1 genome copy numbers were measured using qRT-PCR. *p* = 0.0005. Data shown in **a**, **c**–**f**, **h**–**j**, **l** are from one representative experiment of at least 3 biological independent experiments (mean ± SD, *n* = 2 independent samples in **a**, **c**, **e**, **f**, **h**–**j**, and *n* = 3 independent samples in **d**, **l**). Two-tailed Student’s *t*-test was used to analyze statistical significance. *p < 0.05; **p < 0.01; ***p < 0.001 versus the control groups. Source data are provided as a Source data file.

To further substantiate the biological role of PCBP2 in cGAS-STING signal transduction, we used the CRISPR-Cas9 method to generate a *Pcbp2*-knockout L929 cell line and examined whether deletion of *Pcbp2* affects cGAS-mediated antiviral signaling. Wild-type and *Pcbp2-*deficient cells from two different clones were transfected with HT-DNA, and the results of qRT-PCR assays showed that the transcriptional levels of *Ifnb1* and *Ifit1* stimulated by HT-DNA were remarkably increased in *Pcbp2*^*–/–*^ cells compared with wild-type control cells (Fig. 4a and Fig. S5a). Moreover, the levels of TBK1 and IRF3 phosphorylation induced by HT-DNA transfection were also enhanced in *Pcbp2*^*–/–*^ cells compared with *Pcbp2*^*+/+*^ cells under the same experimental conditions (Fig. 4b). We also found that loss of *Pcbp2* significantly increased the mRNA expression of *Ifnb1* following HSV-1 infection in a time-independent manner (Fig. 4c). Moreover, rescue experiments revealed that the restored expression of Pcbp2 could reverse the enhanced *Ifnb1* expression induced by HT-DNA transfection in Pcbp2-deficient cells (Fig. 4d). Consistently, we found that PCBP2 knockout in human THP-1 cells also enhanced the mRNA levels of *IFNB1* and *IFIT1* induced by HSV-1 infection (Fig. 4e, f) and increased levels of TBK1 and IRF3 phosphorylation induced by HT-DNA (Fig. 4g). These data further support the notion that PCBP2 functions as a negative regulator in cGAS-mediated antiviral signaling pathway.

To study the physiological function of PCBP2 in vivo, we next tried to generate *Pcbp2*-knockout mice using the CRISPR-Cas9 method. However, we failed to obtain *Pcbp2*^*−/−*^ mice after the intercrossing of heterozygotes, which was consistent with the previous study^34^. Next, we aimed to isolate *Pcbp2*^*−/−*^ MEFs from 13.5-day-old embryos. We noted that the majority of *Pcbp2*^*−/−*^ embryos had died at day 13.5, but fortunately, two surviving *Pcbp2*^*−/−*^ embryos were obtained and dissected to isolate *Pcbp2*^*−/−*^ MEFs for further functional assays (Fig. S5b). As shown in Fig. S5c–e, the level of *Ifnb1* mRNA was significantly enhanced in *Pcbp2*^*−/−*^ MEFs compared with wild-type cells following stimulation with HSV-1, HT-DNA, or VACV70, which derives from the vaccinia virus DNA. Intriguingly, we found that the abundance of *Ifnb1, Ifit1*, and *Cxcl10* mRNA in *Pcbp2*^*+/−*^ cells was also markedly higher than that in *Pcbp2*^*+/+*^ cells following HSV-1 infection (Fig. 4h–j). Consistently, the levels of TBK1 and IRF3 phosphorylation induced by HSV-1 were enhanced in Pcbp2^+/–^ cells compared with their *Pcbp2*^*+/+*^ counterparts (Fig. 4k). Furthermore, we tested whether PCBP2 was involved in controlling DNA virus amplification. As shown in Fig. 4l, the HSV-1 genomic DNA copy number was substantially lower in *Pcbp2*^*+/−*^ cells than that in wild-type cells. Collectively, our findings emphasize that PCBP2 negatively regulates cGAS-STING antiviral signaling.

### PCBP2 modulates cGAS-STING signaling by specifically targeting cGAS

**Fig. 5:**
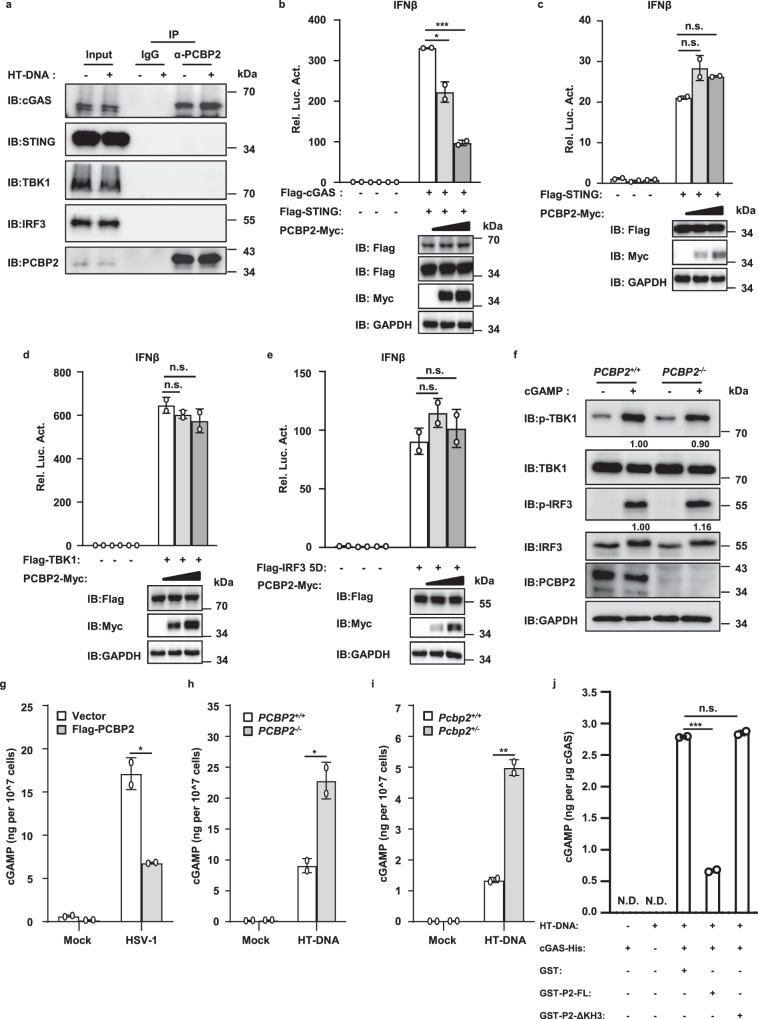
PCBP2 specifically targets cGAS to antagonize its enzyme activity. **a** THP-1 cells were transfected with or without HT-DNA (2 μg/ml), and cell lysates were subjected to Co-IP with a PCBP2 antibody or control IgG, followed by immunoblotting. **b**–**e** HEK293T cells were transfected with IFNβ-Luc and expression vectors encoding cGAS and STING (**b**), STING (**c**), TBK1 (**d**), or IRF3-5D (**e**), together with the increased amount of PCBP2 or an empty vector. After 24 h of transfection, the cells were lysed for luciferase reporter assays (upper panel) and immunoblotting assays (lower panels). **b** *p* = 0.0258; *p* = 0.0004 in sequence. **f** *PCBP2*^*+/+*^ and *PCBP2*^*−/−*^ THP-1 cells were permeabilized with Perfringolysin O (PFO; 300 ng/ml), then treated with or without cGAMP (0.2 μg/ml) for 4 h, followed by immunoblotting. **g** THP-1 cells were infected with a lentivirus expressing PCBP2 or an empty vector for 48 h, followed by infection with HSV-1 for 9 h. Cytoplasmic cGAMP was extracted and quantified with a cGAMP ELISA kit. *p* = 0.0155. **h** PCBP2-knockout and control THP-1 cells were transfected with HT-DNA for 6 h and then harvested to measure the abundance of cGAMP with a cGAMP ELISA kit. *p* = 0.0256. **i** *Pcbp2*^*+/+*^ and *Pcbp2*^*+/−*^ MEFs were transfected with HT-DNA for 6 h, and the cGAMP abundance in cytoplasmic extracts was measured with a cGAMP ELISA kit. *p* = 0.0027. **j** In vitro enzyme activity assays of cGAS were conducted by incubating recombinant human cGAS protein and GST, GST-PCBP2, or GST-PCBP2-△KH3 with HT-DNA (50 ng/µl) at 37 °C for 2 h. The mixtures were then incubated with benzonase at 37 °C for 30 min, heated at 95 °C for 5 min, followed by centrifugation. The heat-resistant supernatants were used to determine the abundance of cGAMP using L929-ISRE cells. *p* < 0.0001. P2: PCBP2. Data shown in **b**–**e**, **g**–**j** are from one representative experiment of at least 2 biological independent experiments (mean ± SD, *n* = 2 independent samples). Two-tailed Student’s *t*-test was used to analyze statistical significance. *p < 0.05; **p < 0.01; ***p < 0.001; n.s. not significant versus the control groups. Source data are provided as a Source data file.

We next aimed to better understand the molecular mechanism by which PCBP2 antagonizes the cGAS-mediated antiviral response. To determine whether cGAS is a specific target of PCBP2, we performed Co-IP experiments to examine if PCBP2 can interact with other known components in the cGAS-STING pathway, such as STING, TBK1, and IRF3. First, we co-transfected HEK293T cells with PCBP2 and cGAS, STING, TBK1, or IRF3 and found that PCBP2 only pulled down cGAS but not the other components (Fig. S6a). Second, we used an anti-PCBP2 antibody and performed endogenous Co-IP experiments in THP-1 cells in the presence or absence of HT-DNA stimulation. Only cGAS, but not the other components, was found to associate with PCBP2 (Fig. 5a). In addition, we observed that HT-DNA transfection increased the association between cGAS and PCBP2 (Fig. 5a). Thus, these findings suggest that PCBP2 likely specifically targets cGAS, thereby antagonizing cGAS-STING signaling activity.

Our results described thus far support an intriguing model that PCBP2 negatively regulates the cGAS-STING signaling pathway likely by specifically targeting cGAS. To test our model, we next investigated whether PCBP2 overexpression affected the activation of components downstream from cGAS, including STING, TBK1, and IRF3. In contrast to its role in antagonizing the activation of cGAS, PCBP2 failed to influence the activation of STING, TBK1, or IRF3-5D (a constitutively active form of IRF3) (Fig. 5b–e). In particular, we found that overexpression of PCBP2 did not affect the levels of IRF3 and TBK1 phosphorylation induced by the ectopic expression of STING (Fig. S6b). Collectively, these data suggest that PCBP2 functions upstream of STING to regulate cGAS-STING signaling.

Given that cGAS catalyzes the synthesis of cGAMP, which functions as a second messenger by binding to and activating STING, and PCBP2 functions upstream of STING, we next examined whether PCBP2 was involved in regulating cGAMP-STING signaling. The results of reporter assays demonstrated that cGAMP treatment remarkably increased IFNβ promoter activation in HEK293T cells stably expressing STING. However, PCBP2 overexpression had no effect on the activation of the IFNβ promotor induced by cGAMP treatment (Fig. S6c), suggesting that cGAMP activates STING through a PCBP2-independent mechanism. To confirm our observation, we measured the levels of phosphorylated TBK1 and IRF3 induced by cGAMP in wild-type and PCBP2-deficient THP-1 cells and found that loss of PCBP2 had no effect on the increased levels of phosphorylated TBK1 and IRF3 induced by cGAMP treatment compared with control cells (Fig. 5f). These results indicated that PCBP2 functions upstream of cGAMP-STING to negatively regulate cGAS-cGAMP-STING signaling.

### PCBP2 attenuates the enzymatic activity of cGAS to balance cGAS signaling

Because cGAS is a synthase involved in the production of cGAMP, we reasoned that PCBP2 targets cGAS likely by negatively regulating its enzymatic activity, thus decreasing cGAMP production. To test this hypothesis, we first used a 2′-3′-cGAMP ELISA kit to measure the levels of cGAMP in wild-type and PCBP2-overexpressed THP-1 cells induced by HSV-1 infection. cGAMP levels were significantly reduced in PCBP2-overexpressing cells compared with control cells, suggesting that PCBP2 reduces cGAS-mediated cGAMP production in these cells (Fig. 5g). Consistently, PCBP2 deficiency significantly increased the amount of cGAMP induced by HT-DNA transfection in THP-1 cells (Fig. 5h). In addition, we measured the levels of cGAMP in wild-type and *Pcbp2* knockdown Raw 264.7 cells and found that knockdown of *Pcbp2* also augmented the level of cGAMP induced by HT-DNA (Fig. S6d). We also observed that the abundance of cGAMP induced by HT-DNA was enhanced in *Pcbp2*^*+/−*^ MEFs compared with that in control cells (Fig. 5i). Moreover, we purified PCBP2 wild-type, PCBP2-ΔKH3, and cGAS from *E. coli* and performed in vitro cGAMP synthesis assays. We found that PCBP2-FL reduced cGAS enzymatic activity, whereas the PCBP2-ΔKH3 mutant did not exhibit this reducing ability (Fig. 5j). Collectively, these data suggest that PCBP2 acts upstream of cGAMP and modulates cGAS signaling by regulating its enzymatic activity.

### PCBP2 reduces cGAS enzymatic activity by antagonizing its condensation

**Fig. 6:**
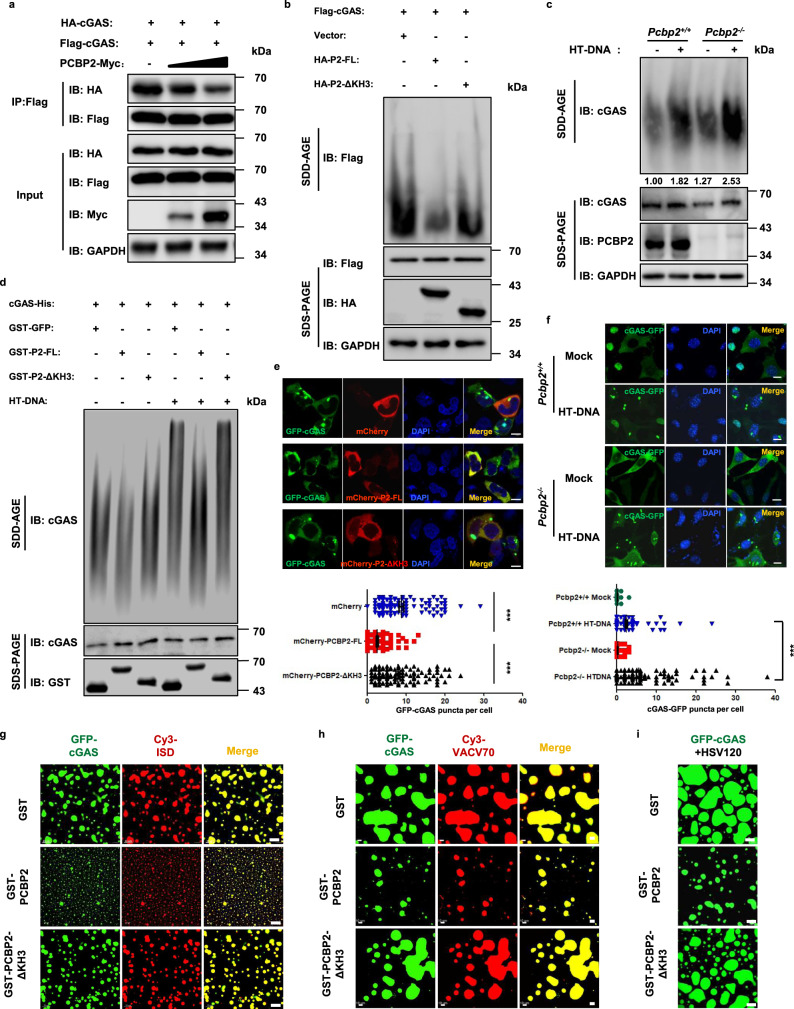
PCBP2 attenuates the cGAS condensation. **a** HEK293T cells were transfected with the indicated expression plasmids. After 24 h of transfection, cell lysates were subjected to Co-IP with anti-Flag beads, followed by immunoblotting. **b** HEK293T cells were transfected with the indicated expression plasmids. After 24 h of transfection, cell lysates were resolved with SDD-AGE or SDS-PAGE, followed by immunoblotting. **c** *Pcbp2*^*+/+*^ and *Pcbp2*^*−/−*^ L929 cells were transfected with HT-DNA (2 μg/ml) for 6 h, and the cell lysates were resolved with SDD-AGE or SDS-PAGE, followed by immunoblotting. **d** Recombinant cGAS and PCBP2 wild-type or its KH3-deleted mutant proteins were incubated with or without HT-DNA (200 μg/ml) at 25 °C for 1.5 h and then resolved by SDD-AGE and SDS-PAGE, followed by immunoblotting. **e** HEK293A cells were transfected with GFP-cGAS, together with mCherry-tagged wild-type PCBP2 or its KH3-deleted mutant or mCherry for 24 h. The cells were fixed, stained with DAPI (blue), and observed by confocal microscopy. Scale bars, 10 μm. The number of cGAS granules per cell was quantified (bottom), at least 100 cells from each group were analyzed. *p* < 0.0001; *p* < 0.0001 in sequence. P2: PCBP2. **f** *Pcbp2*^*+/+*^ and *Pcbp2*^*−/−*^ L929 cells stably expressing mouse cGAS-GFP were transfected with or without HT-DNA (2 μg/ml) for 6 h. The cells were then fixed, stained with DAPI (blue), and observed by confocal microscopy. Scale bars, 10 μm. The number of cGAS granules per cell was quantified (bottom), at least 100 cells from each group were analyzed. *p* < 0.0001. **g**–**i** Recombinant GFP-cGAS proteins (5 μM) were incubated with 2 μM of GST-tagged wild-type PCBP2 or its KH3-deleted mutant or GST proteins in the presence of 2.5 μM Cy3-ISD (**g**), Cy3-VACV70 (**h**), or HSV120 (**i**) for 5 min. Confocal images are representative of cGAS condensates of all fields. Scale bars, 10 μm. Data shown in **e**, **f** are from one representative experiment of at least three independent experiments (mean ± SD, *n* = 107 cells in **e**, and *n* = 133 cells in **f**). Two-tailed Student’s *t*-test was used to analyze statistical significance. ***p < 0.001. Source data are provided as a Source data file.

Next, we investigated how PCBP2 regulates the enzymatic activity of cGAS. Previous studies have shown that DNA binding induces cGAS conformational changes and that cGAS can form dimers and undergo aggregation, which plays an important role in regulating its enzymatic activity^6,22^. Our experiments described above demonstrated that PCBP2 interacted with cGAS and decreased its enzymatic activity. Thus, we hypothesized that PCBP2 likely modulated cGAS enzyme activity by regulating cGAS condensation. To test this hypothesis, we conducted the following experiments. First, HA-cGAS and Flag-cGAS were co-transfected with PCBP2 or an empty vector into HEK293T cells, and then Co-IP assays were conducted to examine whether PCBP2 affected the self-association of cGAS. As shown in Fig. 6a, the self-association of cGAS was affected by PCBP2 overexpression. Second, we performed semi-denaturing detergent agarose gel electrophoresis (SDD-AGE) assays to detect protein aggregates. As shown in Fig. 6b, we found that overexpressed cGAS could form high molecular weight (HMW) condensates, whereas co-expression of wild-type PCBP2, but not KH3-deleted mutant, reduced the levels of cGAS condensates in HEK293T cells. Conversely, *Pcbp2* knockout enhanced cGAS condensates induced by HT-DNA treatment in L929 cells (Fig. 6c). Similar results were obtained in *PCBP2* knockout THP1 cells following HSV-1 infection (Fig. S7a). In addition, we conducted an in vitro cGAS condensates assay and found that wild-type PCBP2, but not KH3-deleted mutant, significantly attenuated cGAS condensates regardless of the presence or absence of HT-DNA treatments (Fig. 6d). Third, we conducted immunostaining experiments and found that overexpression of PCBP2, but not KH3-deleted mutant, significantly reduced the formation of cGAS granules in HEK293A cells (Fig. 6e). Conversely, cGAS formed substantially more granules in *Pcbp2*^*−/−*^ L929 cells than in control cells after stimulation of HT-DNA (Fig. 6f). Consistently, more cGAS granules were observed in *PCBP2*^*−/−*^ THP-1 cells compared with those in wild-type cells following ISD treatment or HSV-1 infection (Fig. S7b, c). To obtain more stringent evidence that support the idea of PCBP2-regulated cGAS condensation, we used the PROTAC technology to rapidly degrade PCBP2 protein and then traced the in vivo behavior of cGAS granules upon HT-DNA stimulation. We generated PCBP2-mCherry-FKBP12^F36V^ knock-in HeLa cells, in which the mCherry-FKBP12^F36V^ was fused to the downstream of the endogenous PCBP2. The cells with stably expressed GFP-cGAS were transfected with HT-DNA, and then treated with dTAG^V^-1 or dTAG^V^-1-NEG (negative control)^35^. To trace the in vivo behavior of cGAS granules, we performed the live-cell imaging assays. As shown in Fig. S7d-f and Movie S1, 2, the treatment with the dTAG^V^-1 (but not dTAG^V^-1-NEG) not only rapidly degraded PCBP2 protein, but it also caused a dynamical increased size of cGAS granules. Moreover, we employed Split GFP system using GFP1-10-cGAS and cGAS-GFP11 to examine whether PCBP2 affected cGAS self-association. As shown in Fig. S7g, h, GFP1-10-cGAS and cGAS-GFP11 could bind together to form cellular foci and HSV-1 infection enhanced the intensity of foci, whereas overexpression of PCBP2 significantly reduced the intensity of cGAS foci in the presence or absence of HSV-1 infection. Taken together, these findings suggest that PCBP2 modulated cGAS condensation.

A previous study showed that cGAS could form liquid-like droplets after binding to DNA, which plays an important role in regulating cGAS activity^24^. To test whether PCBP2 affects cGAS condensation through phase separation, we purified GFP-cGAS and GST-PCBP2 proteins and conducted in vitro protein phase separation analysis^24^. First, we examined whether PCBP2 formed phase separation with DNA, since PCBP2 has been reported to bind to DNA^27^. We observed that, unlike cGAS, PCBP2 did not form liquid droplets in the presence of HSV120 (Fig. S8a). Next, we incubated different amounts of GST-PCBP2 or GST control protein together with mixture of cGAS and HSV120, found that GST-PCBP2 but not GST reduced the DNA-induced condensation of cGAS in a dose-dependent manner (Fig. S8b). We also observed that liquid droplet formation of cGAS with DNA needed a higher concentration of cGAS in the presence of GST-PCBP2, when compared to control (in the presence of GST) (Fig. S8c). In addition, we found that PCBP2 reduced the formation of cGAS droplets, regardless of the stimulation of ISD, VACV70 or HSV120 (Fig. S8d). Moreover, we observed that wild-type PCBP2 but not KH3-deleted mutant had the ability of reducing the formation of cGAS-DNA droplets induced by ISD45 (Fig. 6g), VACV70 (Fig. 6h), or HSV120 (Fig. 6i). Intriguingly, in contrast to mCherry (control) or mCherry-PCBP2-ΔKH3 proteins that were evenly distributed in solution, we found that mCherry-PCBP2 proteins accumulate on the surround of cGAS-DNA droplets, consequently reducing the size of cGAS-DNA droplets compared to the control (Fig. S8e, Movie S3–5). It would be interesting to understand how PCBP2 condensates with cGAS to form multilayered structures in future study. Collectively, these findings suggest that PCBP2 modulates cGAS condensation through association with cGAS.

## Discussion

cGAS functions as a cytosolic sensor to detect the pathogenic DNA of viruses^36^, bacteria^37^, or damaged self-DNA^38^. After binding to DNA, cGAS becomes activated and catalyzes the synthesis of cGAMP, which binds to STING to active downstream signaling, subsequently inducing the production of IFNs and inflammatory cytokines^4,39^. cGAS-cGAMP-STING-mediated signaling is tightly regulated to maintain innate immune system homeostasis and prevent the overproduction of IFNs, which can be detrimental to the host and even lead to the development of autoimmune diseases^7^. Previous studies have reported several negative regulators of cGAS, such as AKT kinase^8^, p62^21^, Beclin1^20^, Caspase 1/3^17,18^, Gasdermin D^19^, and TTLL6/4^10^. For example, AKT was found to phosphorylate cGAS, subsequently decreased its enzymatic activity^8^. Autophagy protein P62 regulates cGAS stability through autophagic degradation^21^, and Beclin1 suppresses cGAS activity by interacting with cGAS^20^. Caspase 1/3 were found to cleave cGAS^17,18^, while Gasdermin D reduces cGAS activation through driving K^+^ efflux^19^. TTLL6/4 were found to glutamylate cGAS, subsequently suppressed its activity^10^. Although these studies have made significant progress in understanding the regulatory mechanism of cGAS activity, the processes that balance the activity of cGAS to avoid harmful overreaction still remain largely unknown. In this study, we found that PCBP2 maintained proper cGAS signaling by targeting cGAS. Mechanistically, we found that PCBP2 attenuated the enzyme activity of cGAS to reduce cGAMP production by antagonizing cGAS condensation. This study provides a regulatory mechanism that is important for balancing the activity of cGAS, thus maintaining cellular homeostasis.

A previous study showed that PCBP2 mRNA and protein levels were induced by SeV infection^32^. PCBP2 was mainly localized in the nuclei of untreated cells but could translocate to the cytosol, co-localize with MAVS in mitochondria upon SeV infection, and recruit the E3 ligase AIP4 to catalyze MAVS ubiquitination, thereby targeting it for degradation^32^. We tested whether PCBP2 exhibited a similar function upon HSV-1 infection. We observed no apparent enhancement of *PCBP2* mRNA, whereas *cGAS* and *IFNB1* mRNA were significantly increased following HSV-1 infection in THP-1 cells (Fig. S9a–c). However, immunostaining assays revealed that PCBP2 could translocate to the perinuclear region, where it showed a punctate distribution in HeLa cells after transfection with HT-DNA (Fig. S9d). Similarly, we observed that PCBP2 translocated to the cytosol when cells were infected with HSV-1 or transfected with cGAS (Fig. S9d and e). These results suggested that PCBP2 can translocate to the cytoplasm and target cGAS to reduce antiviral signaling upon DNA virus infection. How PCBP2 translocates to the cytosol following virus infection is an interesting question that requires further investigation. Of note, PCBP1 is also a member of the poly(C)-binding protein (PCBP) family and has been shown to play a negative role in modulating MAVS-mediated signaling by targeting MAVS for degradation. In this study, we found that PCBP1 played a positive role in regulating cGAS-mediated signaling, which is consistent with the previous findings^40^ (Fig. S10a, b). It would be interesting to address the issue of different functions between PCBP1 and PCBP2 in regulating innate immune response to DNA and RNA virus infection in the future.

Given both cGAS and PCBP2 are nucleic acid-binding proteins, we examined whether the cGAS-PCBP2 interaction occurs in a nucleic acid-dependent manner, and we found that treatment of DNase or RNase did not affect the interaction between PCBP2 and cGAS (Fig. S11a, b). Moreover, we found that the addition of polyI/C or HT-DNA had no effects on PCBP2-cGAS interaction (Fig. S11b). Consistent results were obtained when co-IP assays were performed using purified cGAS-His and GST-PCBP2 from *E. coli* (Fig. S11c). Taken together, our findings suggest that the cGAS-PCBP2 interaction occurs in a nucleic acid-independent manner.

cGAS condensates is important for its enzyme activity. Upon binding to DNA, cGAS can undergo aggregation^6,22^ and form liquid droplets in vitro and in vivo^24^. Both DNA binding and self-association of cGAS are important for the formation of the cGAS oligomeric complex^6^; however, the mechanisms that dynamically regulate this complex to maintain an appropriate innate immune response are still unclear. In this study, we obtained extensive evidence supporting that PCBP2 reduces cGAS condensates. First, Co-IP experiments suggest that PCBP2 regulated cGAS self-association. Second, SDD-AGE assays reveal that PCBP2 overexpression reduced cGAS condensates in HEK293T cells, whereas PCBP2 deficiency enhanced cGAS condensates induced by HSV-1 infection and HT-DNA stimulation. Third, immunostaining assays showed that PCBP2 overexpression reduced the formation of cGAS granules, whereas PCBP2 knockout enhanced the formation of cGAS granules induced by HSV-1 infection and HT-DNA. Forth, the live-cell imaging assays traced the in vivo behavior of cGAS granules upon HT-DNA stimulation and showed that the treatment with the dTAG^V^-1 not only rapidly degraded PCBP2 protein, but it also caused a dynamically increased size of cGAS granules. Fifth, split GFP assays showed that PCBP2 overexpression significantly reduced the intensity of cGAS foci in the presence or absence of HSV-1 infection. Finally, in vitro phase separation assays supported that PCBP2 remarkably reduced the DNA-induced liquid phase condensation of cGAS. Collectively, our findings bring insight into understanding the mechanism underlying the dynamic regulation of the cGAS condensates.

## Methods

**Ethics statements**

All animal studies were carried out in strict accordance with the recommendations in the Guide for the Care and Use of Laboratory Animals of the Ministry of Science and Technology of the People’s Republic of China. The protocols for animal studies were approved by the Committee on the Ethics of Animal Experiments of the Institute of Zoology, Chinese Academy of Sciences (Beijing, China) (approval number: IOZ15001).

### Cell culture

HEK293T (GNHu17), HeLa (TCHu187), L929 (GNM28), THP-1 (TCHu57), and RAW264.7 (TCM13) cells were obtained from the Shanghai Cell Bank of the Chinese Academy of Sciences (Shanghai, China). HEK293T, HeLa, L929, and RAW264.7 cells were maintained in Dulbecco’s modified Eagle’s medium (DMEM, Gibco, Cat# C11995500BT) supplemented with 10% fetal bovine serum (Biological Industries, Cat# 04-001-1ACS), 1% penicillin, and 1% streptomycin (Invitrogen, Cat# 15140122). MEFs from wild-type and mutant mice were generated from 13.5-day-old embryos and cultured in complete DMEM containing 1 mM sodium pyruvate (Invitrogen, Cat# 11360070), 10 mM L-glutamine (Invitrogen, Cat# 25030081), 10 mM β-mercaptoethanol (Invitrogen, Cat# 21985-023), and 1% nonessential amino acids (Gibco, Cat# 11140). THP-1 cells were maintained in RPMI 1640 medium (Gibco, Cat# C11875500BT) supplemented with 10% fetal bovine serum (Biological Industries, 04-001-1ACS), 1% penicillin, 1% streptomycin (Invitrogen, Cat# 15140122), 10 μM β-mercaptoethanol (Invitrogen, Cat# 21985-023), and 5 mM HEPES (Sigma, Cat# H4034).

### Plasmids

For construction of Flag-tagged or hemagglutinin (HA)-tagged mammalian expression plasmids, cDNA of PCBP2, cGAS, STING, TBK1, or IRF3 was amplified using TransStart^®^ FastPfu DNA Polymerase (TransGen Biotech, Cat# AP221-01), and inserted into pCDH or pcDNA3.0 vector. For construction of histidine (His)-tagged cGAS or glutathione S-transferase (GST)-tagged PCBP2 bacterial expression plasmids, cDNAs were subcloned into pET-28a or pGEX4T-1 vector, respectively. To construct mammalian expression plasmids encoding GFP-tagged cGAS, mCherry-tagged PCBP2, spGFP1-10-V5-cGAS, PCBP2-2×spGFP11, cGAS-2×spGFP11, DNA fragments encoding the tag protein, PCBP2 or cGAS were amplified and assembled to linear pCDH vector using ClonExpress MultiS One Step Cloning Kit (Vazyme, Cat# C113-02); and DNA fragments encoding GFP-cGAS or mCherry-PCBP2 were assembled to linear pET-28a vector to generate the bacterial expression plasmids. PCBP2 and cGAS mutants were generated by PCR-based mutagenesis using 2x Phanta Max Master Mix (Vazyme, Cat# P525-03). IFNβ-, ISRE-luciferase (Luc) reporter plasmids were kindly provided by Dr. Hongbing Shu^41^, and NF-κB-Luc reporter plasmid was generously provided by Dr. Zhijian Chen^42^.

### Antibodies

Rabbit anti-phospho-IRF3 (Cat# 4947, 1:1000), rabbit anti-IRF3 (Cat# 4302, 1:1000), rabbit anti-phospho-TBK1 (Cat# 5483, 1:1000) and rabbit anti-cGAS antibodies (Cat# 15102, Cat# 79978, Cat# 31659 (Mouse specific), 1:1000) were from Cell Signaling Technology. Rabbit anti-TBK1 (Cat# ab40676, 1:1000) was from Abcam. Rabbit anti-PCBP2 (Cat# 15070-1-AP, 1:1000) was from Proteintech. Mouse anti-PCBP2 (Cat# sc-101136, 1:1000) was from Santa Cruz Biotechnology. Mouse anti-glyceraldehyde-3-phosphate dehydrogenase (GAPDH) (Cat# KM9002, 1:4000), mouse anti-α-Tubulin (Cat# KM9007, 1:8000), and mouse anti-HA (Cat# KM8004, 1:1000) antibodies were from Sungene Biotechnology. Rabbit anti-Flag (Cat# F7425, 1:1000) antibody was from Sigma. Mouse anti-Flag (Cat# M185-3, 1:1000), rabbit anti-HA (Cat# M132-3, 1:1000), rabbit anti-Myc (Cat# 562, 1:1000), mouse anti-Myc (Cat# M192-3, 1:1000) antibodies were from MBL. Mouse anti-V5 antibody (Cat# YM3005, 1:1000) was from ImmunoWay Biotechnology Company. Rabbit anti-PCBP1 (Cat# A1044, 1:1000) antibody was from ABclonal Technology. Mouse/Rabbit anti-cGAS antibody was prepared in our laboratory, the cGAS antibody was generated by immunizing mice or rabbits with purified human cGAS full-length from *E. coli*.

### DNA oligonucleotides

All DNA oligonucleotides were synthesized by Tsingke Biological Technology Company. For Cy3-labeled ISD or VACV70, the sense strand was modified at the 5’ end; the antisense strand was not modified. The sequences of oligonucleotides used in this study were as follows:

ISD45:

5′-TACAGATCTACTAGTGATCTATGACTGATCTGTACATGATCTACA-3′.

VACV70:

5′-CCATCAGAAAGAGGTTTAATATTTTTGTGAGACCATGGAAGAGAGAAAGAGATAAAACTTTTTTACGACT-3′.

HSV120:

5′-AGACGGTATATTTTTGCGTTATCACTGTCCCGGATTGGACACGGTCTTGTGGGATAGGCATGCCCAGAAGGCATATTGGGTTAACCCCTTTTTATTTGTGGCGGGTTTTTTGGAGGACTT-3′.

### Luciferase reporter analysis and transfection

HEK293T cells were transfected using calcium phosphate transfection method or polyethylenimine. A Renilla reporter plasmid and firefly luciferase reporter plasmid encoding IFNβ-Luc, NF-κB-Luc, or ISRE-Luc were co-transfected with the indicated expression plasmids. An empty control plasmid was used in the same experiment to ensure that the same amount of total DNA was transfected. Cells were lysed to measure luciferase activity. Firefly luciferase activity was normalized to Renilla activity. All reporter assays were repeated at least three times, the results of other two repeated experiments are shown in Fig. S12.

### Co-IP and immunoblotting analysis

Cells were lysed in lysis buffer (20 mM Tris-HCl, pH 7.5, 150 mM NaCl, 0.5% Triton X-100, 10% glycerol, 1 mM EDTA) supplemented with a complete protease inhibitor cocktail (Roche, Cat# 4693132001). Clarified cell lysates were incubated with anti-Flag M2 agarose beads (Sigma Aldrich, Cat# A2220) for 4 h at 4 °C. The immunoprecipitated complexes were washed with lysis buffer containing 300 mM NaCl three times and subjected to immunoblotting with the indicated antibodies. For endogenous IP, cell lysates were incubated with a cGAS or PCBP2 antibody overnight at 4 °C, followed by further incubation with protein A/G beads (Pierce, Cat# 53133) for 2 h, followed by immunoblotting. For immunoblotting analysis, the samples were separated by SDS-PAGE, and transferred to BioTrace NT Nitrocellulose Transfer Membrane (Pall Corporation, Cat# 66485), which were then incubated with specific primary antibodies and secondary HRP-conjugated antibodies (KPL, Cat# 074-1806 or Cat# 074-1506, 1:5000). Chemiluminescent detection kit (Thermo Scientific, Cat# 34580) was used for signal visualization.

### Identification of cGAS-interacting proteins by mass spectrometry

HEK293A cells were infected with lentivirus expressing SFB-tagged cGAS or an empty vector for 48 h, then infected with HSV-1 for 10 h (MOI = 3) or mock-infected. The cells were lysed with lysis buffer (20 mM Tris-HCl, pH 7.5, 150 mM NaCl, 1% NP-40, 0.5% DOC, 0.1% SDS, 10% glycerol, 1 mM EDTA, 1 mM EGTA) containing a complete protease inhibitor cocktail, followed by centrifugation at 20,000 × *g* for 10 min at 4 °C. The supernatants were subjected to immunoprecipitation using S-protein agarose (Millipore, Cat# 69704). Immunoprecipitates were separated by SDS-PAGE, and the gel was stained with Coomassie brilliant blue. The entire lane was cut into 2-mm gel slices, digested with Trypsin, and subjected to LC-MS/MS assays using an Orbitrap Elite mass spectrometer (Thermo Fisher Scientific). The mass spectrometry data were analyzed using Thermo Proteome Discovery (version 2.3), and tandem mass spectra were searched against the UniProt-*Homo sapiens* database.

### cGAS-interacting protein heatmap and network assembly

Specificity filtering on abundance count was conducted using the SAINT (Significance Analysis of INTeractome) algorithm^43^ in SAINTexpress (version 3.6.1) software^44,45^, and a value of 0.9 was used as a specificity threshold for the IPs conducted in HEK293A-SFB-cGAS. Proteins with SAINT values less than 0.9 were removed from the finalized cGAS-interacting protein lists, and three keratins were further removed from the list as contaminants (Supplementary Dataset 1). In order to directly compare the enrichment values of different proteins in cGAS-interacting protein, abundance counts of each protein were all scaled between −2 and 2, and the remaining proteins were displayed in the heatmap plot.

The specificity-filtered proteins (Supplementary Dataset 1) were submitted to the STRING database^46,47^. Through STRING, network edges were incorporated from a combination of evidence sources, including previously published experiments, databases, co-expression, neighborhood, gene fusion and co-occurrence, and a high confidence (0.7) was set as the minimum required interaction score. The network data were downloaded from STRING and imported into Cytoscape (version 3.8.2) to visualize the cGAS interaction network^48^, through Cytoscape, the average enrichment of abundance count in either the Mock or HSV-1-infected conditions were depicted as the color of each node, and the SAINT scores were indicated as the size of each node.

### Expression and purification of recombinant proteins in *E. coli*

Constructs encoding human cGAS, GFP-tagged cGAS, mCherry-tagged PCBP2, and its mutant were cloned into the pET-28a vector carrying a C-terminal 6*His tag. GST-tagged full-length human PCBP2 and its mutant were inserted into a pGEX-4T-1 vector containing an N-terminal GST-tag. The plasmids were transformed into the BL21 *E. coli* strain. The fusion proteins were purified from the cell lysates using Ni-Sepharose beads (GE Healthcare, Cat# 17-5318-02) or Glutathione-Sepharose beads (GE Healthcare, Cat# 17-0756-01) in accordance with the manufacturer’s protocols.

### Immunofluorescence staining and live-cell imaging assay

HeLa, HEK293A, L929, or THP-1 cells were seeded on gelatin-coated glass coverslips and then transfected or infected as indicated. The cells were fixed with 4% paraformaldehyde for 15 min, permeabilized, and blocked with PBS containing 0.2% Triton-X-100 and 5% BSA for 25 min at room temperature (RT), and then incubated with the primary antibody, followed by the secondary antibody. The cells were washed with PBST (PBS with 0.2% Tween 20) between each step. Images were acquired using a Zeiss LSM 710 META laser scanning confocal system or an ANDOR CR-DFLY-505 confocal microscope. For quantitative co-localization analysis, images were captured using a Nikon A1 confocal microscope. Pearson’s co-localization coefficient was measured using NIS-Elements AR analysis 5.20.00 64-bit software.

For live-cell imaging assays, PCBP2-mCherry-FKBP12^F36V^ knock-in HeLa cells stably expressing GFP-cGAS were grown on a glass-bottom cell culture dish, then transfected with HT-DNA (2 μg/ml). After 3 h of HT-DNA transfection, the cells were treated with dTAG^V^-1 (1 μM) or dTAG^V^-1-NEG (1 μM). The videos were recorded using an ANDOR CR-DFLY-505 confocal microscope equipped with a sCMOS Zyla 4.2 plus camera, and images were analyzed using Imaris software. For Movie processing, Adobe Premiere Pro 2020 was used.

### Quantitative reverse-transcription polymerase chain reaction (qRT-PCR)

Total RNA was isolated using TRIZOL reagent (Invitrogen, Cat# 15596018). cDNA was generated using the SuperScript II First-Strand cDNA Synthesis kit (TianGen Biotech, Cat# KR116). qRT-PCR was conducted in duplicate using a SYBR Green Master Mix (CoWin Biosciences, Cat# CW0957H) on a Bio-Rad CFX Maestro (BIO-RAD) or a Light Cycler 480® (Roche). Relative mRNA levels were normalized to GAPDH or actin mRNA levels in each sample. Relative expression changes were calculated by the 2^−ΔΔCt^ method. Data are shown as the mRNA abundance relative to control groups. All qRT-PCR assays were repeated at least three times, the results of other two repeated experiments are shown in Fig. S12. The primers used were synthesized by Tsingke Biological Technology Company, and the sequences are listed in Supplementary Table1.

### Lentivirus-mediated PCBP2 overexpression and shRNA knockdown

Full-length cDNA encoding human PCBP2 was amplified and inserted into a pCDH-CMV-Puro vector. The lentivirus particles for PCBP2 overexpression were produced by co-transfecting a pCDH-CMV-Puro-PCBP2 construct into HEK293T cells with the packaging plasmids pMD2.G and pAS-MAX. To generate PCBP2-knockdown cells, we used pLKO.1-puro-based lentiviruses expressing specific short hairpin RNAs (shRNAs) against PCBP2. RAW264.7 cells or THP-1 cells were infected with lentiviruses expressing shRNAs against PCBP2 (shPCBP2) or control vector (pLKO), and the cells were selected with puromycin to generate stable PCBP2 knockdown or control pLKO cells. The knockdown efficiency was determined by qRT-PCR or western blotting analysis. The shRNA sequences against human *PCBP2* or mouse *Pcbp2* are as follows:

Human *PCBP2* shRNA-1#:

5′-ACCGGGCATTCCACAATCCATCATTGCTCGAGCAATGATGGATTGTGGAATGCTTTTT-3′

Human *PCBP2* shRNA-2#:

5′-ACCGGCCATGATCCATCTGTGTAGTTCTCGAGAACTACACAGATGGATCATGGTTTTT-3′

Mouse *Pcbp2* shRNA-1#:

5′-ACCGGCCCATCCATAATCCTGCTGTTCTCGAGAACAGCAGGATTATGGATGGGTTTTT-3′

Mouse *Pcbp2* shRNA-2#:

5′-ACCGGTCCTGAGAGAATTATCACTTTCTCGAGAAAGTGATAATTCTCTCAGGATTTTT-3′

### CRISPR/Cas9-mediated PCBP2-knockout cell lines

To generate PCBP2 knockout (*PCBP2*^*−/−*^) cells, lenti-CRISPRv2-sgPCBP2-Puro vectors were constructed in accordance with the method described by Sanjana et al.^49^ and co-transfected with packaging plasmids into HEK293T cells. Two days after transfection, the viruses were harvested and used to infect THP-1 or L929 cells. The infected cells were selected with puromycin (2 μg/ml) for at least 5 days. PCBP2-knockout cells were verified by immunoblotting. *PCBP2* guide RNA sequences were as follows:

Human *PCBP2* sg-s: 5′-CACCG CAGGGTGACCGGGGGTCTAC-3′

Human *PCBP2* sg-as: 5′-AAAC GTAGACCCCCGGTCACCCGTC-3′

Mouse *Pcbp2* sg-s: 5′-CACCG ATCTGTTAAGAAGATGCGCG-3′

Mouse *Pcbp2* sg-as: 5′-AAAC CGCGCATCTTCTTAACAGATC-3′

### CRISPR/Cas9-mediated PCBP2-knockout mice

To generate Pcbp2 knockout C57BL/6J mice, the CRISPR/Cas9-mediated gene deletion system was used. Cas9 mRNA and single-guide RNA targeting *Pcbp2* sequences were co-injected into zygotes to obtain heterozygous mutants. The sequences targeting *Pcbp2* were (5′-3′): ATCTGTTAAGAAGATGCGCG. The mutant was obtained with 8 bp deletion in the fourth exon of Pcbp2, which prematurely terminate protein translation. All mice were housed in a specific pathogen-free animal facility, with controlled temperature (~23 °C), humidity (40–50%), and light/dark cycle (12 h/12 h). Homozygous and heterozygous MEF cells were obtained from 13.5-day-old embryos by breeding 12-week-old male and female heterozygous mice and verified by immunoblotting analysis.

### cGAS enzyme activity assays and cGAMP quantification

For in vitro cGAS reaction assay, recombinant human cGAS protein was mixed with GST-PCBP2 or GST control in the presence or absence of HT-DNA (50 ng/µl) in a low-salt buffer (20 mM HEPES, pH 7.5, 5 mM MgCl_2_, 2 mM ATP, 2 mM GTP) and incubating the mixture at 37 °C for 2 h. The mixtures were incubated with benzonase (Sigma, Cat# E8263, 1 U/μL) at 37 °C for 30 min, then heated at 95 °C for 5 min, followed by centrifugation. The supernatant was incubated with L929-ISRE cells that were permeabilized with digitonin solution (50 mM HEPES pH 7.0, 100 mM KCl, 3 mM MgCl_2_, 85 mM sucrose, 0.1 mM DTT, 0.2% BSA, 1 mM ATP, 0.1 mM GTP, and 10 μg/ml digitonin) at 37 °C for 30 min. The cells were cultured at 37 °C for 12 h and then lysed for luciferase assays. Serial dilutions of cGAMP were used to generate the standard curve for quantifying cGAMP concentrations in the reactions.

For measurement of cGAMP levels in HT-DNA transfected cells, a 2′-3′-cGAMP ELISA kit (Cayman Chemical, Cat# 501700) was used. Briefly, cells were transfected with HT-DNA using Lipofectamine 2000. Four hours after transfection, the cells were harvested and lysed in 100 µl of hypotonic buffer (10 mM Tris-HCl, pH 7.5, 5 mM KCl, and 3 mM MgCl_2_). Cell lysates were heated at 95 °C for 5 min, followed by centrifugation to remove the denatured proteins. The heat-resistant supernatants were used to measure cGAMP abundance. The 2′-3′-cGAMP ELISA kit was used in accordance with the protocol of the manufacturers.

### Measurement of HSV-1 genomic DNA copy numbers

*Pcbp2*^*+/+*^ and *Pcbp2*^*+/–*^ MEFs were infected with HSV-1 (MOI = 0.1) and incubated at 37 °C in serum-free DMEM for 1 h. The cells were washed with warm PBS, cultured in complete DMEM for 18 h, and their genomic DNA was extracted. The HSV-1 genomic DNA copy numbers were determined by qRT-PCR using HSV-1-specific primers with the following sequence: 5′-TGGGACACATGCCTTCTTGG-3′; 5′-ACCCTTAGTCAGACTCTGTTACTTACCC-3′.

### SDD-AGE assay

For cGAS aggregates detection in vivo, cells were transfected or infected as indicated and lysed in lysis buffer (0.5% Triton X-100, 50 mM Tris-HCl, 150 mM NaCl, 10% glycerol), the supernatants were mixed in 1× SDD loading buffer (0.5× TBE, 10% glycerol, 2% SDS, 0.0025% bromophenol blue) and loaded onto a vertical 1.5% agarose gel (1× TBE, 1.5% agarose). Electrophoresis was performed in the 1× TBE running buffer (1× TBE, 0.1% SDS) for 35 min at 4 °C with a constant voltage of 100 V, followed by immunoblotting^50^. For in vitro cGAS aggregates detection, equal amounts (1 μg) of recombinant cGAS protein and recombinant PCBP2 protein or its mutants were incubated in the low salt buffer containing 20 mM HEPES, pH 7.5; 5 mM MgCl_2_; 2 mM ATP and 2 mM GTP with or without HT-DNA (200 μg/ml) at 25 °C for 1.5 h. The reactions were stopped by adding loading buffer (2-mercaptoethanol free) and the samples were resolved by SDD-AGE as above.

### In vitro phase separation assay

Recombinant GFP-cGAS protein, GST, GST-PCBP2, mCherry, mCherry-PCBP2 or its ΔKH3 mutant and the indicated DNA were mixed in a glass-bottom cell culture dish. The mixtures were incubated in the buffer containing 20 mM Tris-HCl, pH 7.5, 150 mM NaCl at RT, and images were captured at the indicated times. Phase-separated droplets were imaged using an ANDOR CR-DFLY-505 confocal microscope equipped with a sCMOS Zyla 4.2 plus camera, and images were analyzed using Imaris software.

### Surface plasmon resonance (SPR) assays

His-tagged cGAS were captured on sensor chip CM5 (carboxymethylated dextran surface). Another blank flow cell was used as reference to correct for instrumental and concentration effect. SPR experiments were performed using Biacore X100 instrument (GE Healthcare) in running buffer containing 20 mM Hepes, pH 7.5, 150 mM KCl, 1.5 mM MgCl_2_ and 0.05% Tween 20 at 25 °C. Full-length GST-PCBP2 or its mutant proteins with increasing concentrations were injected into the cGAS-His probes surface and blank flow cell for 2 min at a flow rate of 30 µl/min, dissociated for 2 min in running buffer. Equilibrium and kinetic constants were calculated by a global fit to 1:1 binding model (Biacore X100 evaluation software).

### Split GFP complementation assay

All plasmids used in split GFP complementation assay were constructed according to a previous study^51^. pCDH-spGFP1-10-V5-cGAS plasmid was constructed by inserting fragment containing spGFP1-10, a V5 tag, and human cGAS into pCDH-CMV-puro vector using a ClonExpress II One Step Cloning Kit (Vazyme, Cat# C112-02); pCDH-cGAS-2×spGFP11 and pCDH-PCBP2-2×spGFP11, were made by inserting fragment containing human cGAS or PCBP2, two copies of spGFP11, and a linker sequences GSGSNGSSGSGGSGGGGSGGSRGGSGGGGSGG into pCDH-CMV-puro vector. After transfection with the indicated constructs, GFP formation in HeLa cells was observed by confocal fluorescence microscopy. Images were analyzed by ImageJ for fluorescence intensity quantification.

### Rapid degradation of FKBP12^F36V^-tagged PCBP2 protein by VHL-recruiting PROTAC with dTAG^V^-1 molecule

PCBP2 protein rapid degradation system was generated by using FKBP12^F36V^-tagged protein with VHL-recruiting dTAG^**V**^-1 molecules in accordance with the method described by Nabet et al.^35^. In brief, to generate CRISPR/Cas9-mediated PCBP2-mCherry-FKBP12^F36V^ knock-in HeLa cells, first, the guide RNA for *PCBP2* knock-in was designed and inserted to LentiCRISPRv2 vector. Then *PCBP2*-specific 5′ and 3′homology arms (500 bp upstream and downstream of *PCBP2* stop codon) to flank the mCherry-FKBP12^F36V^-P2A-Puro cassette was cloned into pcDNA3.1 vector, in which CMV promoter was removed, as donor plasmid by using ClonExpress MultiS One Step Cloning Kit (Vazyme, Cat# C113-02). Then the donor and knock-in sgPCBP2 plasmids were co-transfected into HeLa cells. After 72 h of transfection, the cells were sorted by flow cytometry to harvest mCherry positive cells. The PCBP2-mCherry-FKBP12^F36V^ knock-in HeLa cells were treated with dTAG^V^-1 and dTAG^V^-1-NEG (negative control), that was chemically synthesized by Institute of Biomedical Research, Yunnan University, according to the method described recently^35^, for the indicated time, and analyzed by immunoblotting to examine PCBP2 protein degradation.

The sequences of sgPCBP2 for knock-in are as follows:

sgPCBP2 -s: CACCGTTACAGGCTTTCCTCGGAGA;

sgPCBP2 -as: AAAC TCTCCGAGGAAAGCCTGTAAC

### Statistics and reproducibility

Results of all statistical analyses are shown as mean ± SD. Significant differences between samples under different experimental conditions were performed using two-tailed Student’s *t*-test (GraphPad Prism8). For all tests, *P* values < 0.05 were considered statistically significant. All experiments of immunoblotting, GFP-, Cy3-, and mCherry-fluorescence and immunofluorescence were repeated independently at least twice with similar results, and one representative is shown.

## Supplementary information

Supplementary information

Description of Additional Supplementary Files

Supplementary Figures

Supplementary Dataset1

Supplementary Movie 1

Supplementary Movie 2

Supplementary Movie 3

Supplementary Movie 4

Supplementary Movie 5

Reporting-summary

## Data Availability

The mass spectrometry proteomics data have been deposited to the ProteomeXchange Consortium via the PRIDE partner repository^52^ with the dataset identifier “PXD023597”. All unique biological materials used are available from the authors upon reasonable requests. All other relevant data supporting the key findings of this study are available within the article and its Supplementary Information files or from the corresponding author upon reasonable request. Source data are provided with this paper.

## Source data

Source Data
