# Supplementary material for: PCBP2 maintains antiviral signaling homeostasis by regulating cGAS enzymatic activity via antagonizing its condensation
Source: Nat Commun. 2022 Mar 23;13:1564. doi: 10.1038/s41467-022-29266-9 (PMC8943206; doi:10.1038/s41467-022-29266-9)
Supplement: Supplementary file 1 — Supplementary information [file 41467_2022_29266_MOESM1_ESM.docx]

**Description of Additional Supplementary Files**

**Title: Supplementary Movie 1 and 2: Live cell imaging of the PCBP2 rapid degradation affecting the formation of cGAS-DNA granules**

Description: PCBP2-mCherry-FKBP12^F36V^ knock-in HeLa cells stably expressing GFP-cGAS were transfected with HT-DNA (2μg/ml). After 3 h of transfection, the cells were treated with 1μM dTAGV-1 (Movie S1) or dTAGV-1-NEG (Movie S2). Recording started at 10 min after adding PROTAC molecules and lasted approximately 6 h with 5 min intervals. Scale bars, 10 μm.

**Title: Supplementary Movie 3–5 Time-lapse imaging video of PCBP2 affecting the phase separation of cGAS-DNA**

Description: Liquid droplets formed after mixing 5μM GFP-cGAS with 2μM mCherry (Movie S3), mCherry-PCBP2-FL ((Movie S4) or mCherry-PCBP2-ΔKH3 (Movie S5) at RT in the presence of 2.5μM HSV120. Recording started immediately after mixing and lasted approximately 5 min with 5 s intervals. Scale bars, 10 μm.

**Title: Supplementary Dataset 1 The list of cGAS-interacting protein by mass spectrometry.**

**Description:** The interacting proteins of cGAS were screened by Co-IP coupled with mass spectrometry analysis and the proteins with SAINT score higher than 0.9 using SAINTexpress software were listed.
