# Supplementary material for: PCBP2 maintains antiviral signaling homeostasis by regulating cGAS enzymatic activity via antagonizing its condensation
Source: Nat Commun. 2022 Mar 23;13:1564. doi: 10.1038/s41467-022-29266-9 (PMC8943206; doi:10.1038/s41467-022-29266-9)
Supplement: Supplementary file 3 — Supplementary Figures [file 41467_2022_29266_MOESM3_ESM.pdf]

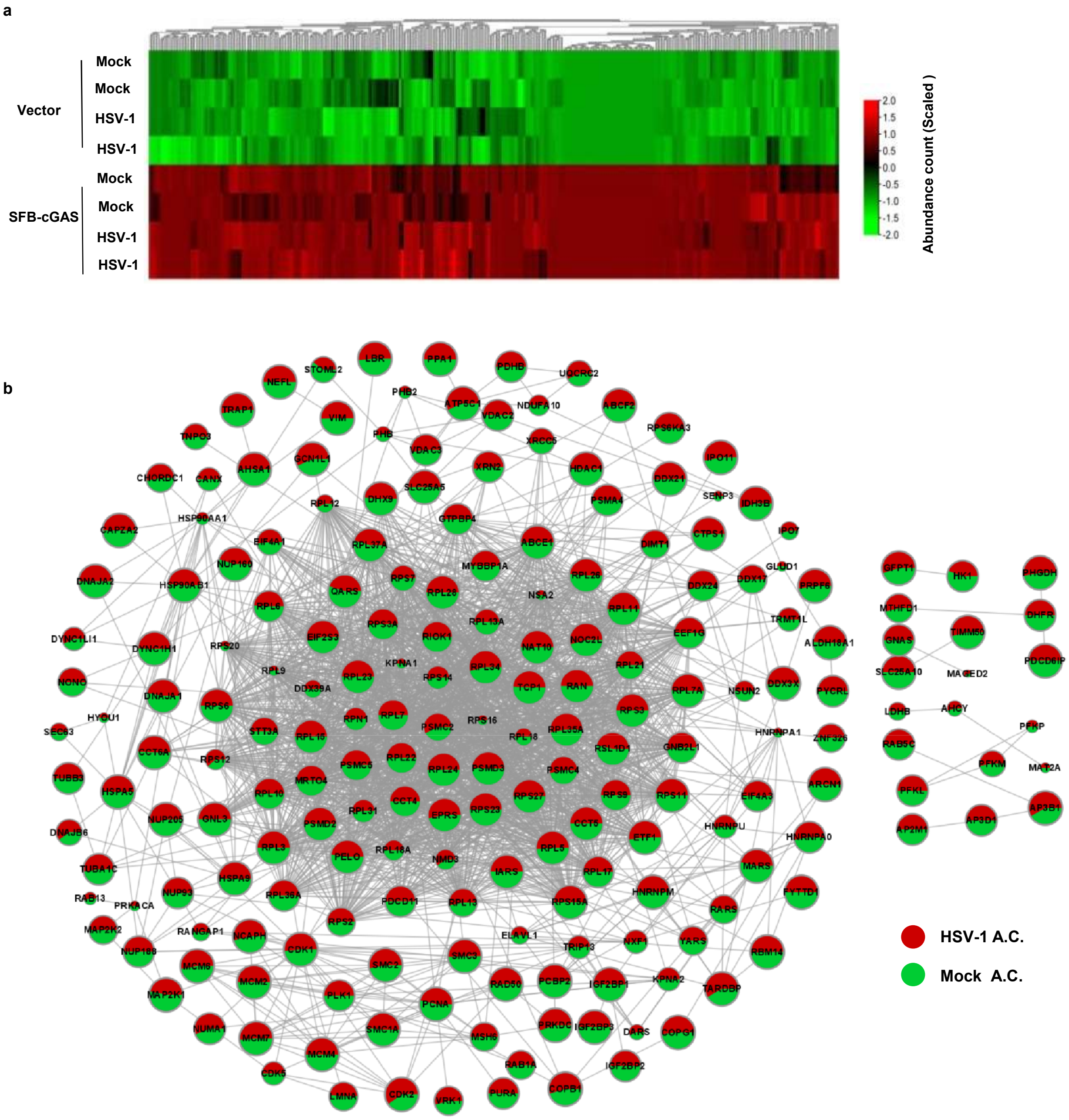

Supplementary Figure 1: The interacting proteins of cGAS were screened by Co-IP coupled with mass spectrometry analysis.

a HEK-293A cells stably expressing SFB-cGAS or an empty vector were infected with HSV-1 for 10 h (MOI=3) or mock infected, followed by immunoprecipitation-mass spectrometry analysis. Significance Analysis of INTeractome algorithm (SAINT) was used for specificity filter on abundance counts, and 258 immunoprecipitates with value higher than 0.9 were retained. After filtering out three keratins, all the remaining 255 immunoprecipitates were shown in the heatmap. The scaled abundance counts enrichment of the proteins were indicated as color of the band (right of the heatmap).

b Proteins with SAINT score higher than 0.9 using SAINTexpress software were assembled in cGAS interaction lists, and then keratin proteins were further removed from the list. Pie charts depict relative abundance count enrichment of the cGAS-interacting protein between mock (green) and HSV-1 (red) infected HEK293A cells stably expressing SFB-cGAS (MOI=3,10 hpi). A.C. Abundance count. The interacting proteins of cGAS were listed in Supplementary Dataset1.

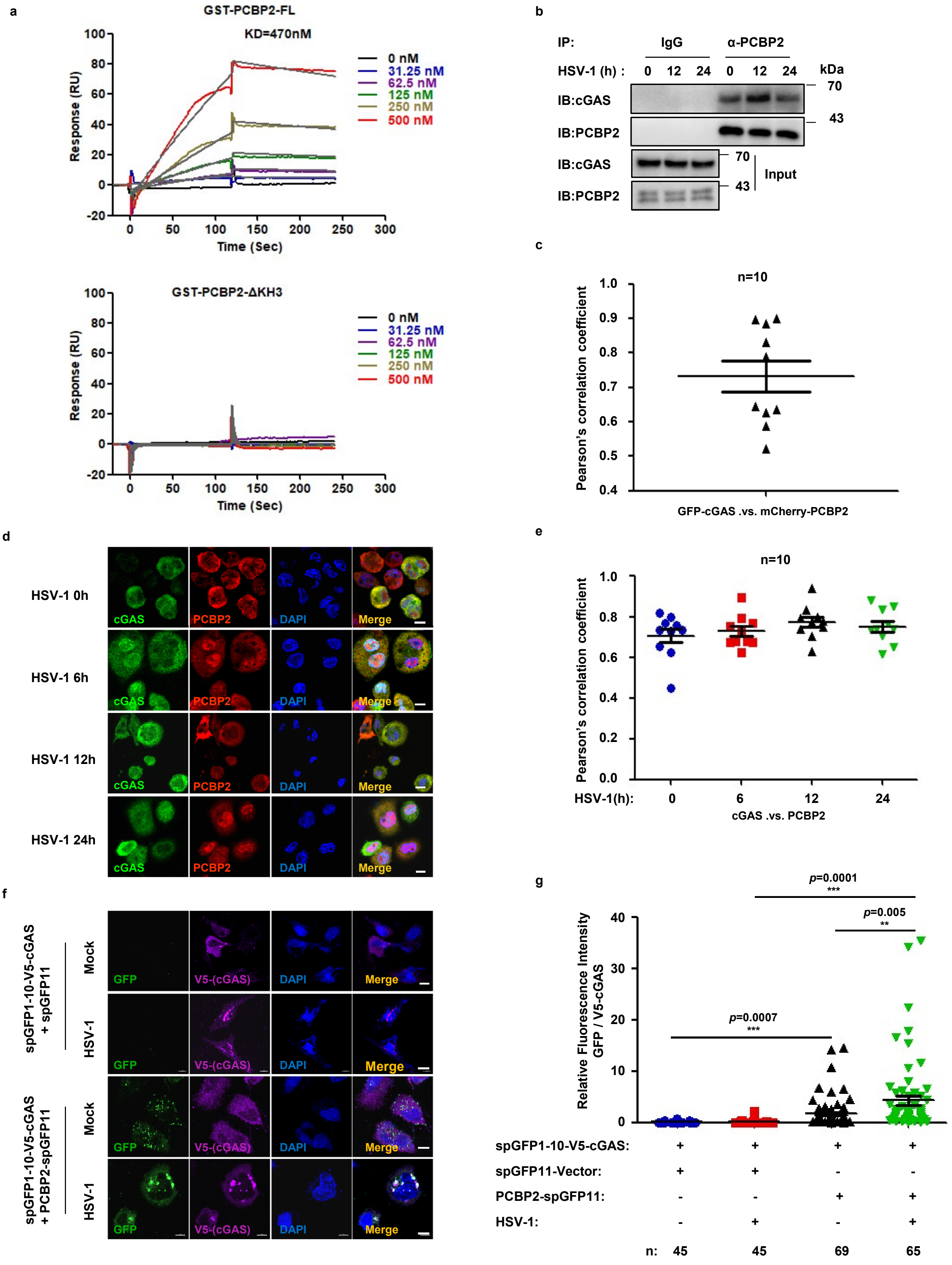
a
GST-PCBP2-FL
KD=470nM
Response (RU)
Time (Sec)
0 nM
31.25 nM
62.5 nM
125 nM
250 nM
500 nM
GST-PCBP2-ΔKH3
Response (RU)
Time (Sec)
0 nM
31.25 nM
62.5 nM
125 nM
250 nM
500 nM
b
IP:
IgG
α-PCBP2
HSV-1 (h) : 0 12 24 0 12 24
kDa
IB:cGAS
70
IB:PCBP2
43
IB:cGAS
70
Input
IB:PCBP2
43
c
n=10
Pearson's correlation coefficient
GFP-cGAS .vs. mCherry-PCBP2
d
HSV-1 0h
HSV-1 6h
HSV-1 12h
HSV-1 24h
cGAS
PCBP2
DAPI
Merge
e
n=10
Pearson's correlation coefficient
HSV-1(h): 0 6 12 24
cGAS .vs. PCBP2
f
spGFP1-10-V5-cGAS + spGFP11
spGFP1-10-V5-cGAS + PCBP2-spGFP11
Mock
HSV-1
GFP
V5-(cGAS)
DAPI
Merge
g
p=0.0001
***
p=0.005
**
p=0.0007
***
Relative Fluorescence Intensity GFP / V5-cGAS
spGFP1-10-V5-cGAS: + + + +
spGFP11-Vector: + + - -
PCBP2-spGFP11: - - + +
HSV-1: - + - +
n: 45 45 69 65

Supplementary Figure 2: PCBP2 associates with cGAS in vitro and in vivo.

a Surface plasmon resonance (SPR) was conducted to measure the interaction between recombinant cGAS-His and GST-PCBP2-FL (upper panel) or its mutant protein (lower panel). Equilibrium and kinetic constants were calculated by a global fit to 1:1 Langmuir binding model. RU, response unit.

b THP-1 STING KO cells were infected with HSV-1 (MOI=10) for the indicated times, cell lysates were then prepared and immunoprecipitated with a PCBP2 antibody or control IgG, followed by immunoblotting.

c Relative co-localization of GFP-cGAS and mCherry-PCBP2 in Fig 1**e** was quantified with Pearson's correlation coefficient by using NIKON NIS-Elements Analysis software 5.20,00. n=10 cells.

d-e THP-1 cells were left untreated or infected with HSV-1 (MOI=10) for the indicated times. Cells were then fixed, stained with cGAS (green), PCBP2 (red) antibody and DAPI (blue), and imaged by confocal microscopy (NIS-Elements AR 5.20,00, Nikon). Scale bars, 10 µm (**d**). Relative co-localization of cGAS and PCBP2 in **d** was quantified with Pearson's correlation coefficient by using NIKON NIS-Elements Analysis software. n=10 cells in (**e**).

f spGFP1-10-V5-cGAS were co-transfected with PCBP2-spGFP11 or spGFP11-Vector (as a negative control) into HeLa cells, followed by stimulation with or without HSV-1 (MOI=5) for 12 h. The cells were fixed and stained with DAPI (blue) and anti-V5 (violet) antibodies. GFP (green) formation was observed by confocal fluorescence microscopy to display the interaction of the indicated proteins. Scale bars, 10 µm . spGFP: split GFP.

g Quantification of GFP to V5-cGAS fluorescence intensity ratio as shown in (**f**). n, cell numbers quantified for each group.

Data shown in **g** are presented as mean values ± SD (n=45, 45, 69, 65 in sequence). Two-tailed Student's t-test was used to analyze statistical significance. ** $p < 0.01$; *** $p < 0.001$ versus the control groups.

Source data are provided as a Source data file.

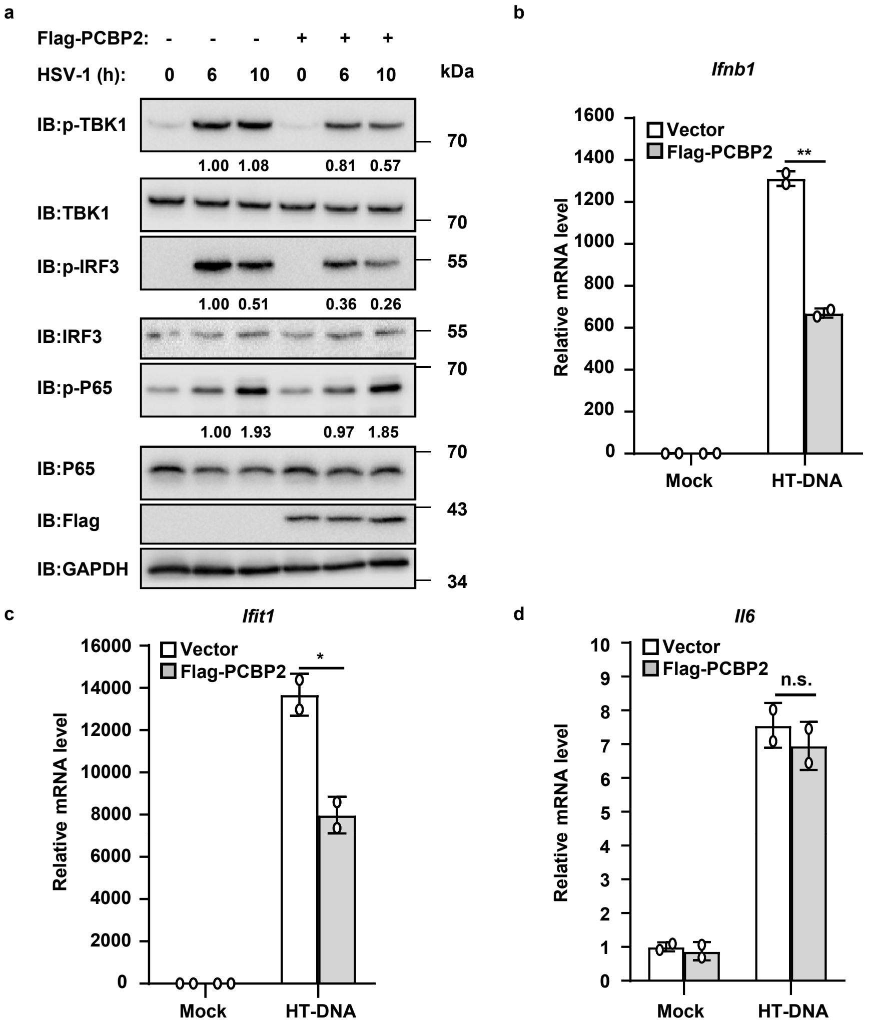

Supplementary Figure 3: PCBP2 overexpression inhibits innate immune response stimulated by HSV-1 and HT-DNA.

a THP-1 cells were infected with a lentivirus expressing PCBP2 or an empty vector for 48 h and then infected with HSV-1 (MOI=10) for the indicated times. Cell lysates were resolved by SDS-PAGE, followed by immunoblotting.

b-d L929 cells were infected with lentivirus expressing Flag-tagged PCBP2 or empty vector for 48 h, followed by HT-DNA (2μg/ml) transfection for 6 h. The cells were harvested to isolate RNA, transcriptional levels of *Ifnb1* (**b**), *Ifit1* (**c**), and *Il6* (**d**) were measured by qRT-PCR analysis. **b** p=0.0022. **c** p=0.0256.

Data shown are from one representative experiment of at least twice independent experiments (mean ± SD, n=2 independent samples in **b-d**). Two-tailed Student's t-test was used to analyze statistical significance. * $p < 0.05$; ** $p < 0.01$; n.s. not significant versus the control groups.

Source data are provided as a Source data file.

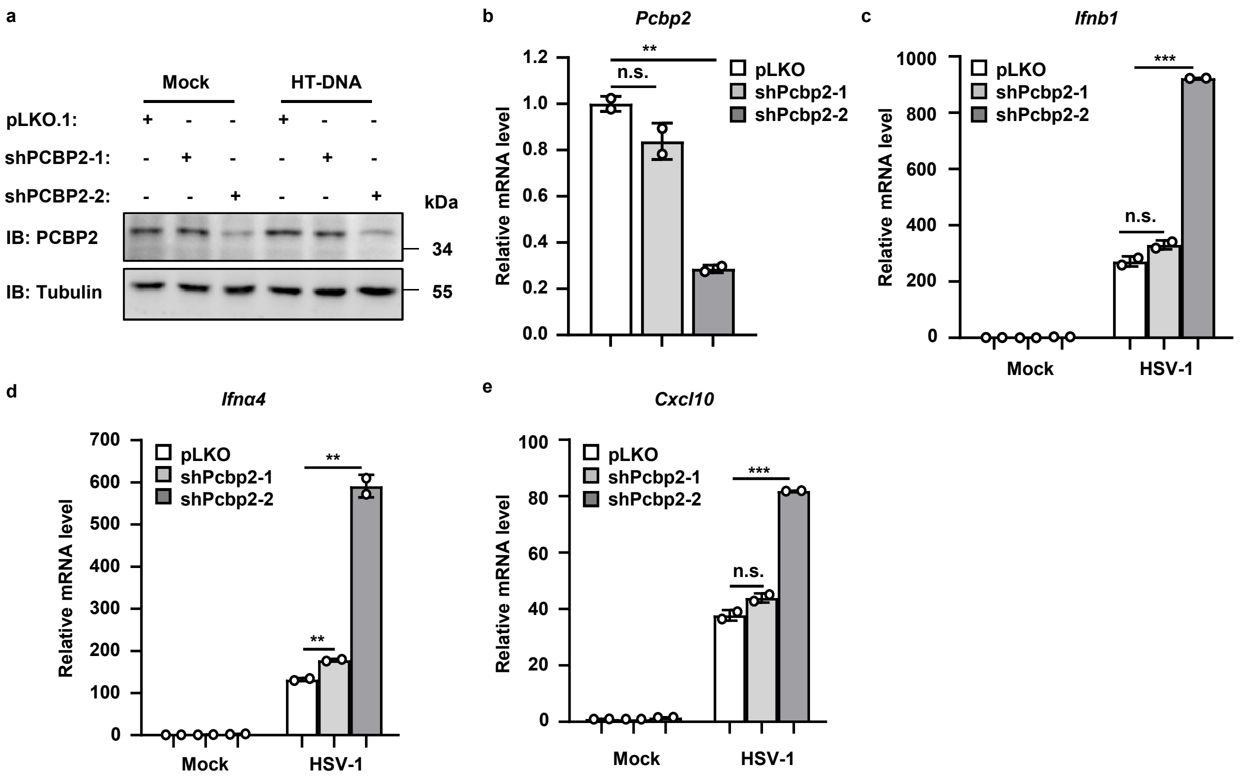

Supplementary Figure 4: Knockdown of PCBP2 potentiates cGAS-STING signaling.

a THP-1 cells were infected with lentivirus-based shRNA targeting PCBP2 or empty vector for 48 h. The cells were transfected with HT-DNA (2μg/ml) for 6 h or mock treated (Mock), lysed, followed by immunoblotting with the indicated antibodies.

b-e RAW264.7 cells were infected with lentivirus-based shRNAs targeting Pcbp2 (shPcbp2) or empty vector for 48 h, then infected by HSV-1 (MOI=5) for 6 h or mock infected. Transcriptional levels of *Pcbp2* (**b**), *Ifnb1* (**c**) *Ifnα4* (**d**) and *Cxcl10* (**e**) were detected by qRT-PCR assays. **b** p=0.0013. **c** p=0.0004. **d** p=0.0051 (shPcbp2-1); p=0.0017 (shPcbp2-2). **e** p=0.0009 (shPcbp2-2).

Data shown are from one representative experiment of at least twice independent experiments (mean ± SD, n=2 independent samples in **b-e**). Two-tailed Student's t-test was used to analyze statistical significance. ** $p < 0.01$; *** $p < 0.001$; n.s. not significant versus the control groups.

Source data are provided as a Source data file.

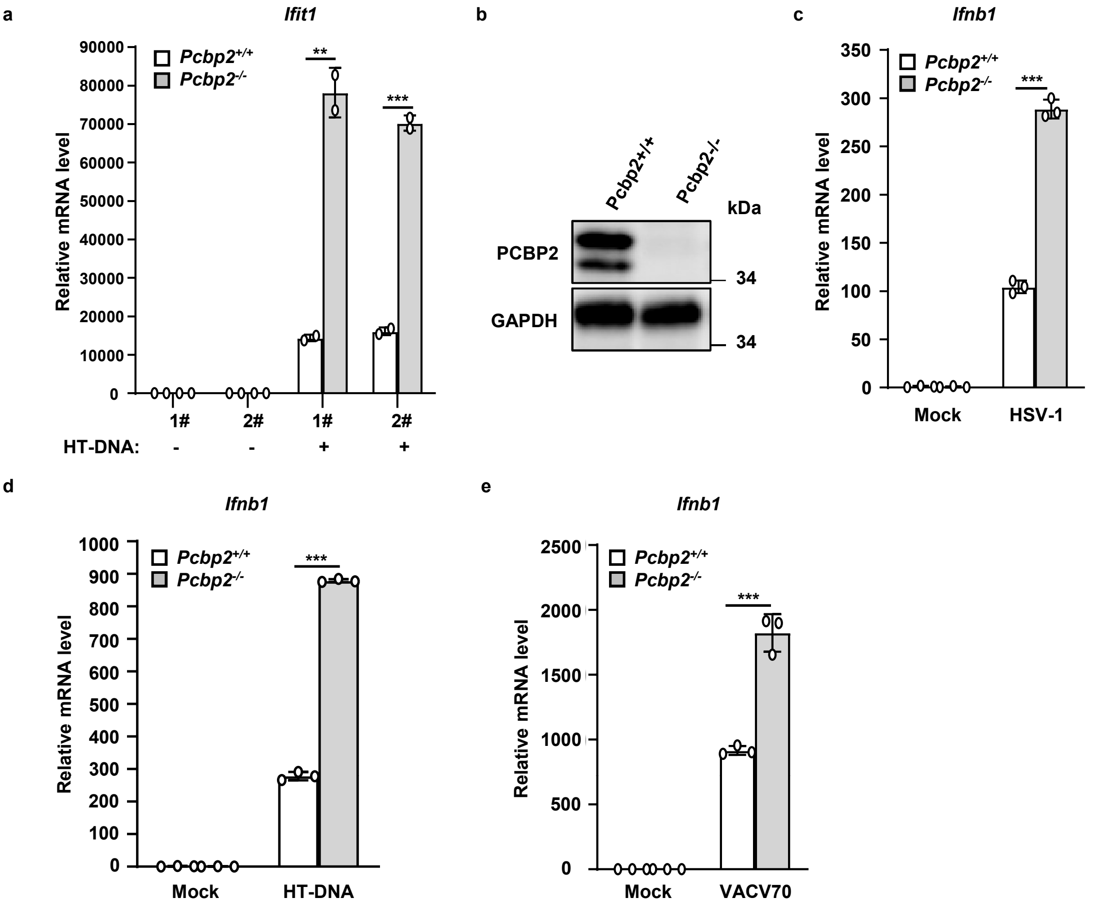

Supplementary Figure 5: PCBP2 knockout increases cGAS-STING signaling.

a Two different clones from wild-type and Pcbp2-deficient L929 cells were transfected with HT-DNA (2μg/ml) for 6 h. The cells were harvested for qRT-PCR analysis to measure the transcriptional levels of *Ifit1*. p=0.0052 (1#); p=0.0008 (2#).

b *Pcbp2*$^{+/+}$ and *Pcbp2*$^{-/-}$ MEFs were lysed for immunoblotting with the indicated antibodies.

c *Pcbp2*$^{+/+}$ and *Pcbp2*$^{-/-}$ MEFs were infected with HSV-1 (MOI=5) for 6 h, then lysed for quantification of mRNA levels of *Ifnb1*. p<0.0001.

d, e *Pcbp2*$^{+/+}$ and *Pcbp2*$^{-/-}$ MEFs were transfected with HT-DNA (2μg/ml) (**d**) or VACV70 (2μg/ml) (**e**) for 6 h, then lysed for quantification of mRNA levels of Ifnb1. **d** p<0.0001. **e** p=0.0005.

Data shown in **a, c-e** are from one representative experiment of at least twice independent experiments (mean ± SD, n=2 independent samples in **a**, and n=3 independent samples in **c-e**).

Two-tailed Student's t-test was used to analyze statistical significance. ** p < 0.01; *** p < 0.001 versus control groups.

Source data are provided as a Source data file.

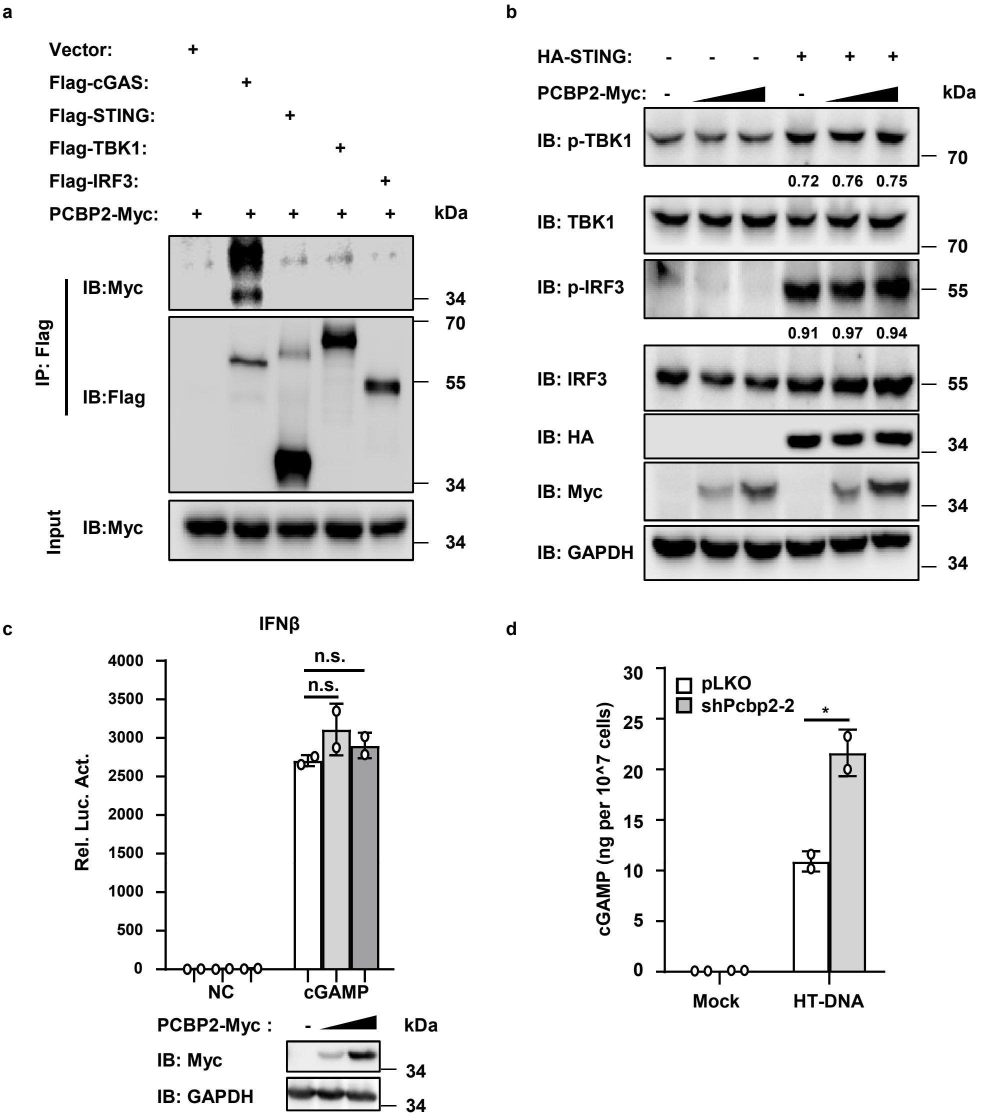

Supplementary Figure 6: PCBP2 specifically targets cGAS to inhibit its activation.

a HEK293T cells were transfected with the indicated plasmids for 30 h. The cells were lysed and immunoprecipitated with Flag beads, followed by immunoblotting.

b HEK293T cells were transfected with the indicated plasmids for 24 h, followed by immunoblotting with the indicated antibodies.

c HEK293T cells stably expressing STING were transfected with the indicated vector together with IFNβ-luc and Renilla which served as an internal control. Twenty-four hours after transfection, the cells were treated with cGAMP for 12 h, then lysed for luciferase reporter assays (upper panel) and immunoblotting assays (lower panels).

d Stable PCBP2 knockdown RAW264.7 cells and control cells were transfected with HT-DNA (2μg/ml) for 6 h and harvested for cGAMP extraction. The abundance of cGAMP was quantitated by cGAMP ELISA kit. p=0.0262.

Data shown in **c**, **d** are from one representative experiment of at least twice independent experiments (mean ± SD, n=2 independent samples. Two-tailed Student's t-test was used to analyze statistical significance. * p < 0.05; n.s. not significant versus the control groups.

Source data are provided as a Source data file.

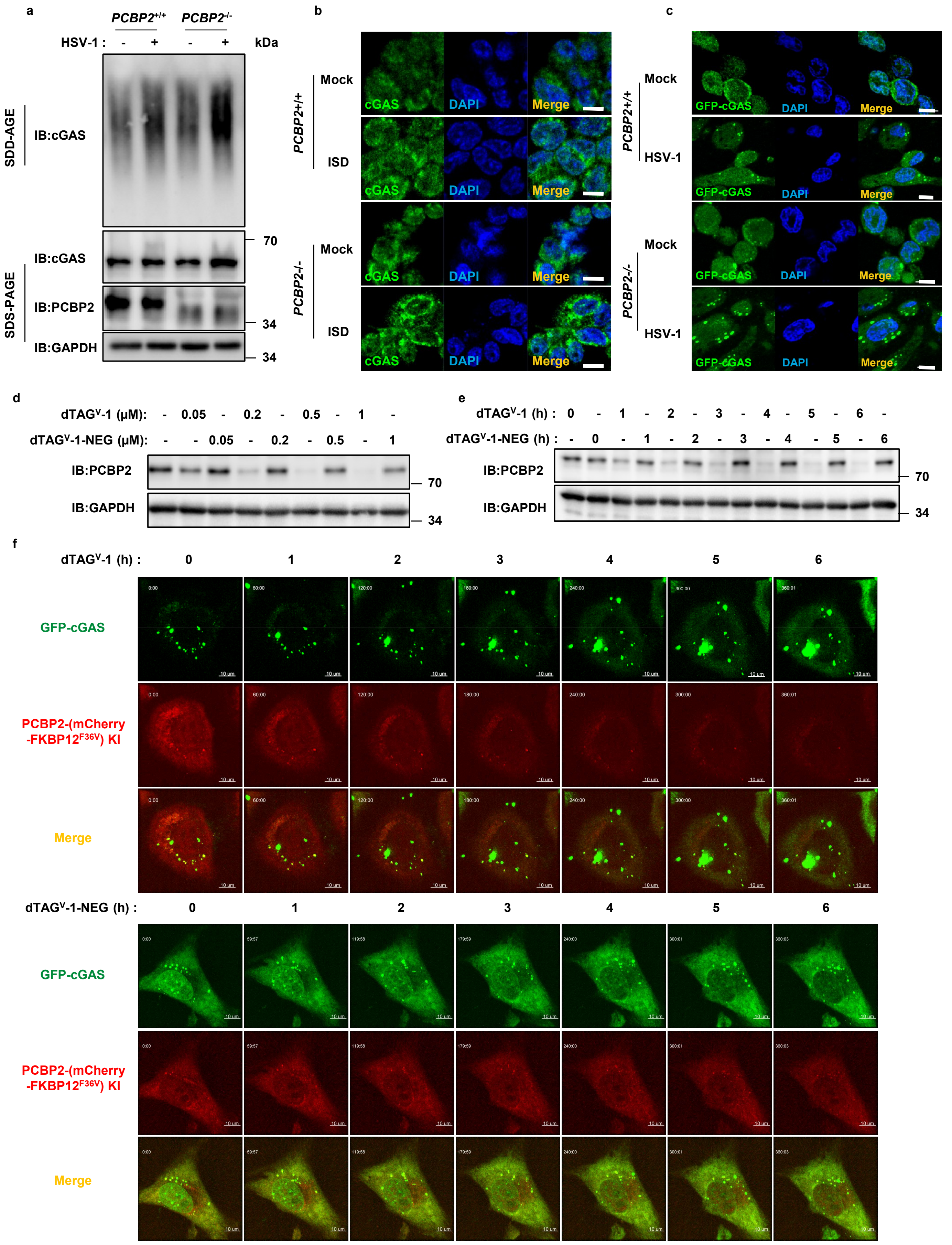
a
PCBP2+/+
PCBP2-/-
HSV-1 : - + - +
kDa
SDD-AGE
IB:cGAS
SDS-PAGE
IB:cGAS
IB:PCBP2
IB:GAPDH
70
34
34
b
PCBP2+/+
PCBP2-/-
Mock
ISD
cGAS
DAPI
Merge
c
Mock
HSV-1
GFP-cGAS
d
dTAGV-1 (μM): - 0.05 - 0.2 - 0.5 - 1 -
dTAGV-1-NEG (μM): - - 0.05 - 0.2 - 0.5 - 1
e
dTAGV-1 (h) : 0 - 1 - 2 - 3 - 4 - 5 - 6 -
dTAGV-1-NEG (h) : - 0 - 1 - 2 - 3 - 4 - 5 - 6
f
dTAGV-1 (h) : 0 1 2 3 4 5 6
PCBP2-(mCherry -FKBP12F36V) KI
dTAGV-1-NEG (h) : 0 1 2 3 4 5 6

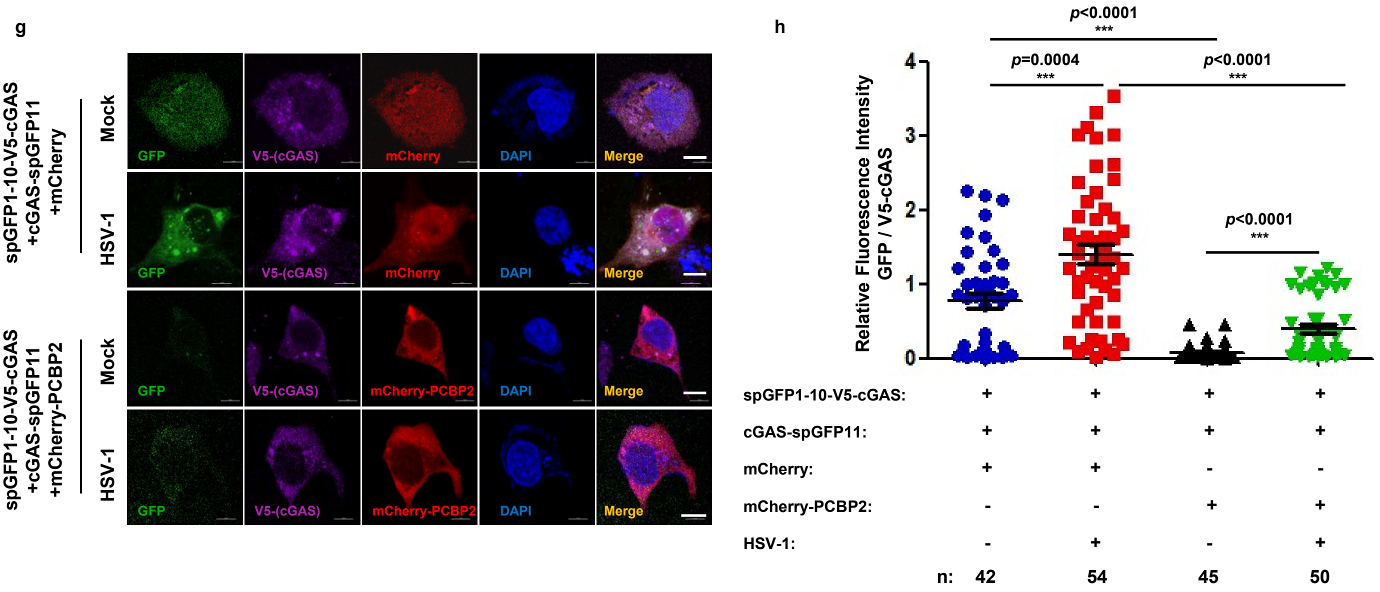

Supplementary Figure 7: PCBP2 attenuates cGAS condensation.

a *PCBP2+/+* and *PCBP2-/-* THP-1 cells were infected with HSV-1 (MOI=5) for 12 h, cell lysates were prepared for SDD-AGE (upper panel) and SDS-PAGE assays (lower panels), followed by immunoblotting.

b *PCBP2+/+* and *PCBP2-/-* THP-1 cells were transfected with or without ISD (2μg/ml) for 6 h. The cells were fixed, stained with cGAS antibody (Sigma, green) and DAPI (blue), and observed by confocal microscopy. Scale bars, 10 μm.

c *PCBP2+/+* and *PCBP2-/-* THP-1 cells stably expressing human GFP-cGAS were infected with HSV-1 (MOI=10) or mock infected for 6 h. Cells were then fixed, stained with DAPI (blue), and observed by confocal microscopy. Scale bars, 10 μm.

d PCBP2-mCherry-FKBP12^{F36V} knock-in HeLa cells were treated with increased concentration of dTAG^{V}-1 or dTAG^{V}-1-NEG for 6 hours and lysed for immunoblotting.

e PCBP2-mCherry-FKBP12^{F36V} knock-in HeLa cells were treated with dTAG^{V}-1 (1μM) or dTAG^{V}-1-NEG (1μM) for the indicated time and lysed for immunoblotting.

f PCBP2-mCherry-FKBP12^{F36V} knock-in HeLa cells were infected with lentivirus expressing GFP-cGAS for 48 h, then transfected with HT-DNA (2μg/ml). After 3 h of transfection, the cells were treated with dTAG^{V}-1 (1μM, upper panels) or dTAG^{V}-1-NEG (1μM, lower panels) for the indicated time and visualized by confocal microscopy. Scale bars, 10 μm. KI: Knock-in.

g HeLa cells were co-transfected with spGFP1-10-V5-cGAS and cGAS-spGFP11, together with mCherry-PCBP2 or mCherry as a negative control, followed by stimulation with or without HSV-1 (MOI=5) for 12 h. The cells were fixed and stained with DAPI (blue) and anti-V5 (violet) antibodies. GFP (green) formation was observed by confocal fluorescence microscopy to display cGAS self-association. Scale bars, 10 μm. spGFP: split GFP.

h Quantification of GFP to V5-cGAS fluorescence intensity ratio as shown in (**g**). n, cell numbers quantified for each group.

Data shown in **h** are presented as mean values ± SD (n=42, 54, 45, 50 in sequence). Two-tailed Student's t-test was used to analyze statistical significance. *** $p < 0.001$ versus the control groups. Data are representative of at least three independent experiments.

Source data are provided as a Source data file.

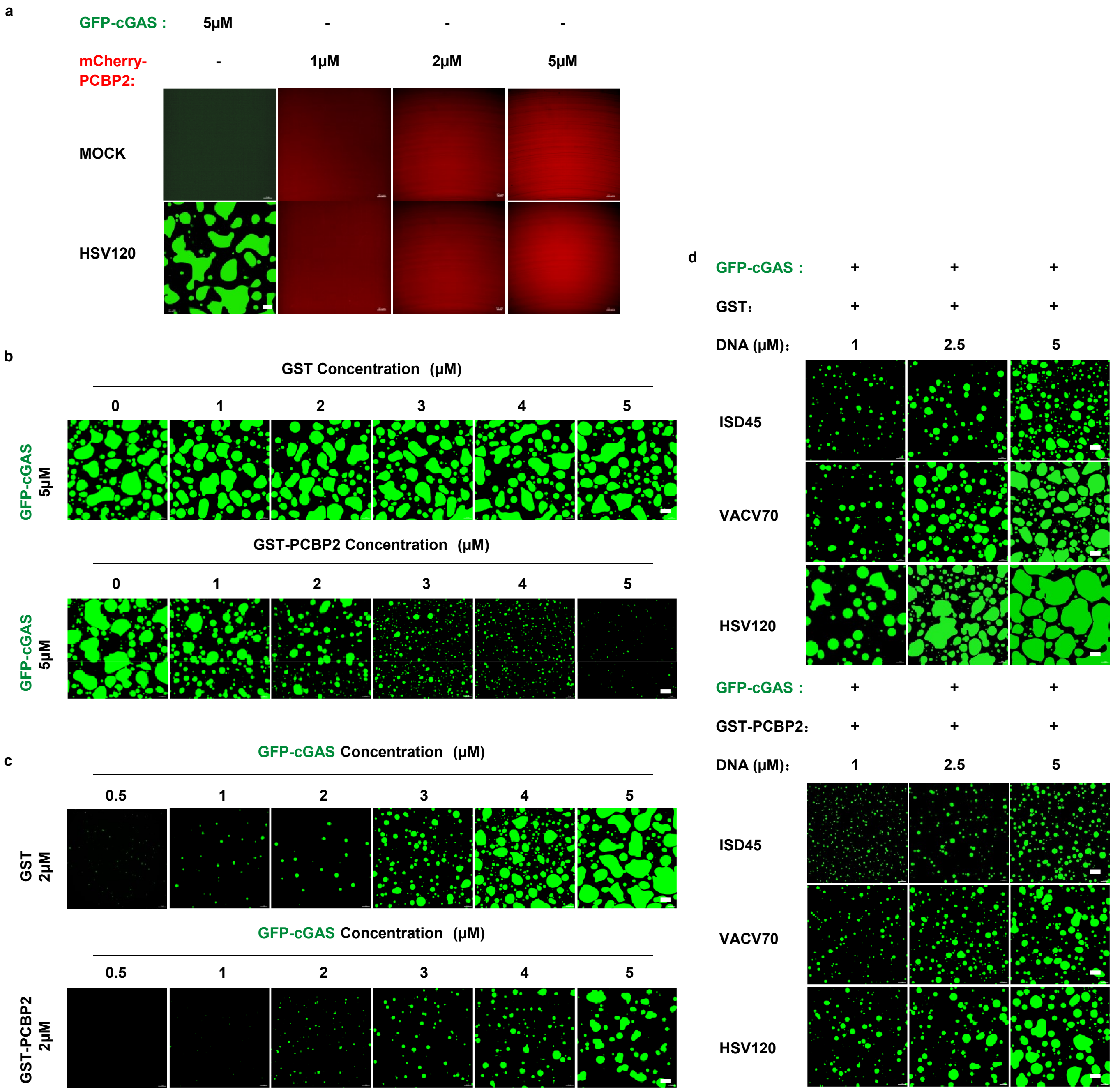
a
GFP-cGAS :
5μM
-
-
-
mCherry-PCBP2:
-
1μM
2μM
5μM
MOCK
HSV120
b
GST Concentration (μM)
0
1
2
3
4
5
GFP-cGAS 5μM
GST-PCBP2 Concentration (μM)
0
1
2
3
4
5
GFP-cGAS 5μM
c
GFP-cGAS Concentration (μM)
0.5
1
2
3
4
5
GST 2μM
GFP-cGAS Concentration (μM)
0.5
1
2
3
4
5
GST-PCBP2 2μM
d
GFP-cGAS :
+
+
+
GST:
+
+
+
DNA (μM):
1
2.5
5
ISD45
VACV70
HSV120
GFP-cGAS :
+
+
+
GST-PCBP2:
+
+
+
DNA (μM):
1
2.5
5
ISD45
VACV70
HSV120

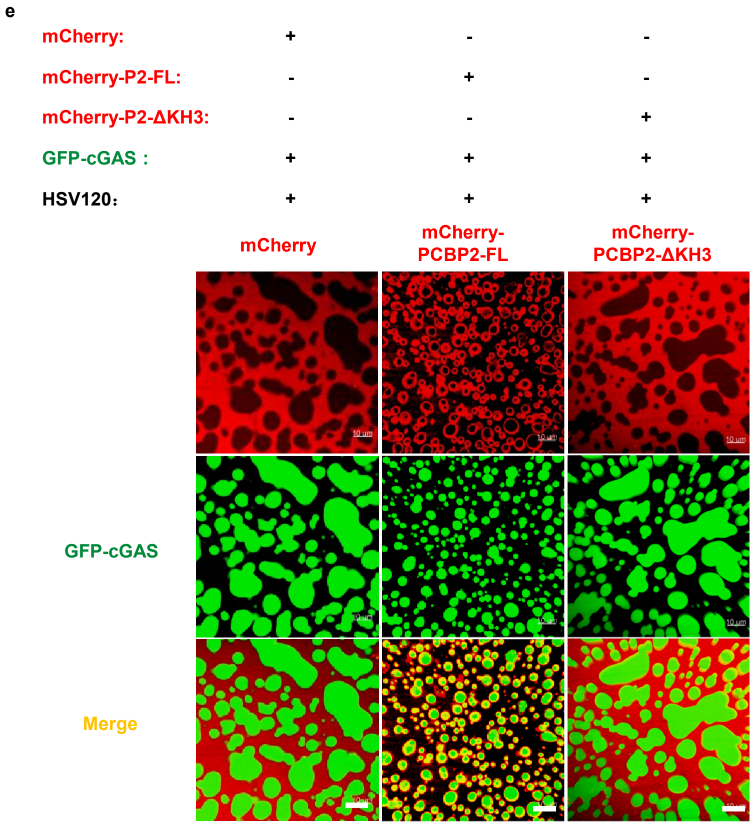

Supplementary Figure 8: PCBP2 reduces cGAS-DNA phase separation.
a Recombinant GFP-cGAS or the increased concentration of mCherry-PCBP2 was incubated with or without HSV120 (2.5μM) for 5 min and observed by confocal microscopy. Scale bars, 10 μm.
b Recombinant GFP-cGAS proteins (5μM) were incubated with the increased concentration of GST-PCBP2 (lower panels) or GST (upper panels) proteins at RT in the presence of HSV120 (2.5μM) for 5 min and observed by confocal microscopy. Scale bars, 10 μm.
c Mixture of GST-PCBP2 (lower panels) or GST (upper panels) with the increased concentrations of GFP-cGAS proteins were incubated at RT in the presence of HSV120 (2.5μM) for 5 min and observed by confocal microscopy. Scale bars, 10 μm.
d Mixture of recombinant GFP-cGAS proteins (5μM) and GST (2μM, upper panels) or GST-PCBP2 (2μM, lower panels) proteins were incubated with the increased concentration of the indicated dsDNA with different length at RT for 5 min and observed by confocal microscopy. Scale bars, 10 μm.
e Recombinant GFP-cGAS proteins (5μM) were incubated with mCherry (2μM), mCherry-PCBP2 (2μM) or mCherry-PCBP2-ΔKH3 (2μM) proteins at RT in the presence of HSV120 (2.5μM) for 5 min and observed by confocal microscopy. Scale bars, 10 μm. P2: PCBP2; FL: Full-length.

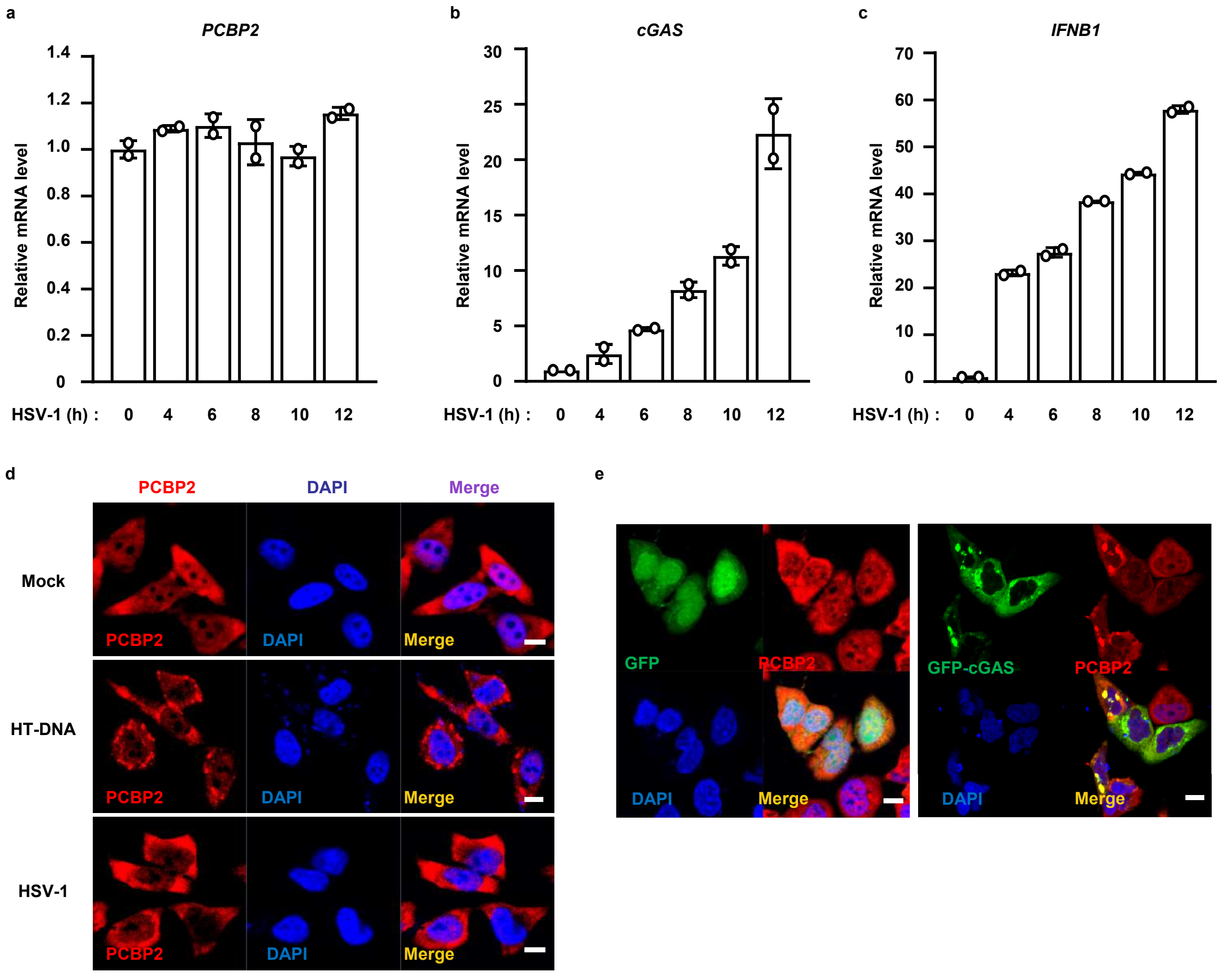

Supplementary Figure 9: PCBP2 mRNA expression is not induced by HSV-1 infection and PCBP2 can translocate to the cytoplasm under different stimulations.

a-c THP-1 cells were infected with HSV-1 (MOI=5) for the indicated times, the cells were then lysed for qRT-PCR analysis to measure the transcriptional levels of *PCBP2* (**a**) *cGAS* (**b**) and *IFNB1* (**c**).

d HeLa cells were left untreated (upper panels), transfected with HT-DNA (2μg/ml) for 6 h (middle panels) or infected with HSV-1 (MOI=2) for 9 h (lower panels). Cells were then fixed, stained with PCBP2 (red) antibody and DAPI (blue), and observed by confocal microscopy. Scale bars, 10 μm.

e HEK293A cells were transfected with GFP or GFP-tagged cGAS. Twenty-four hours after transfection, the cells were fixed, stained with PCBP2 (red) antibody and DAPI (blue), and observed by confocal microscopy. Scale bars, 10 μm.

Data shown in **a-c** are presented as mean values ± SD (n=2 independent samples). Source data are provided as a Source data file.

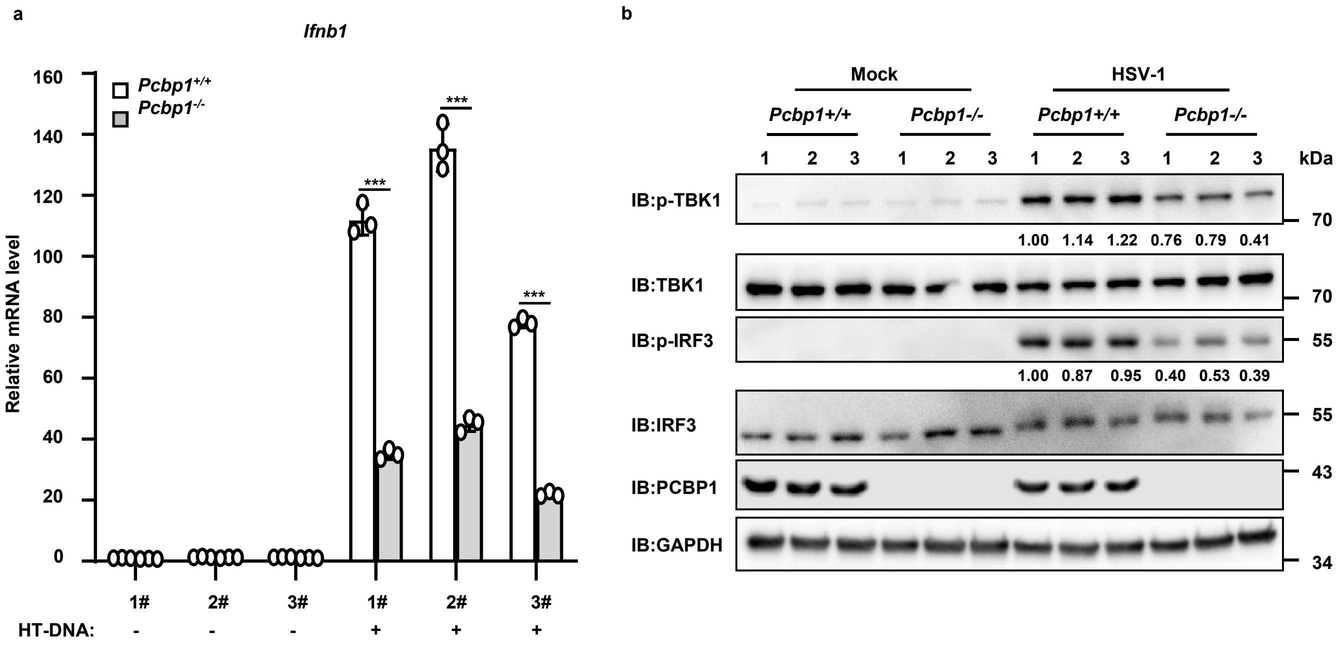

Supplementary Figure 10: Pcbp1 knockout attenuates cGAS-STING signaling.

a Three different clones from wild-type and *Pcbp1*-deficient RAW264.7 cells were transfected with HT-DNA (2μg/ml). Six hours later, the cells were harvested for qRT-PCR analysis to measure the transcriptional levels of *Ifnb1*. *p*<0.0001; *p*<0.0001; *p*<0.0001 in sequence.

b *Pcbp1*$^{+/+}$ and *Pcbp1*$^{-/-}$ RAW264.7 cells were infected with HSV-1 at an MOI of 10 for the indicated times and then lysed for immunoblotting with the indicated antibodies.

Data shown are from one representative experiment of at least twice independent experiments. Data shown in **a** are presented as mean ± SD (n=3 independent samples). Two-tailed Student's t-test was used to analyze statistical significance. *** *p* < 0.001 versus control groups.

Source data are provided as a Source data file.

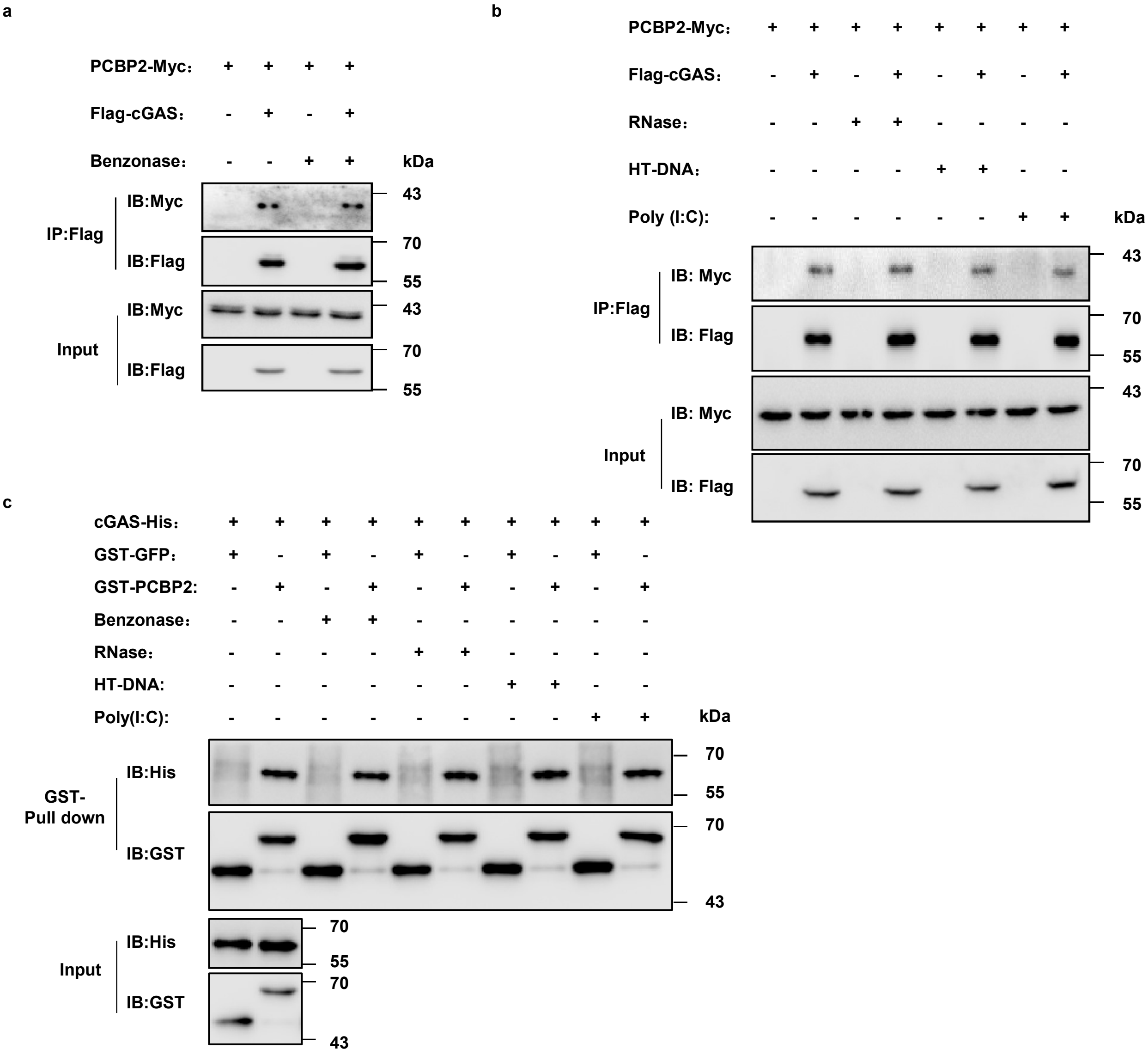

Supplementary Figure 11: Interaction between cGAS and PCBP2 is independent of their nucleic acid binding.

a, b HEK293T cells were transfected with the indicated plasmids for 24 h. The cell lysates were treated as indicated at 37℃ for 20 min and immunoprecipitated with Flag-M2 beads at 4℃, followed by immunoblotting with the indicated antibodies.

c Purified cGAS-His proteins was incubated with GST-PCBP2 or GST-GFP proteins under the different treatments as indicated at 37℃ for 20 min, and then pulled down with glutathione-Sepharose beads at 4℃, followed by immunoblotting.

Source data are provided as a Source data file.

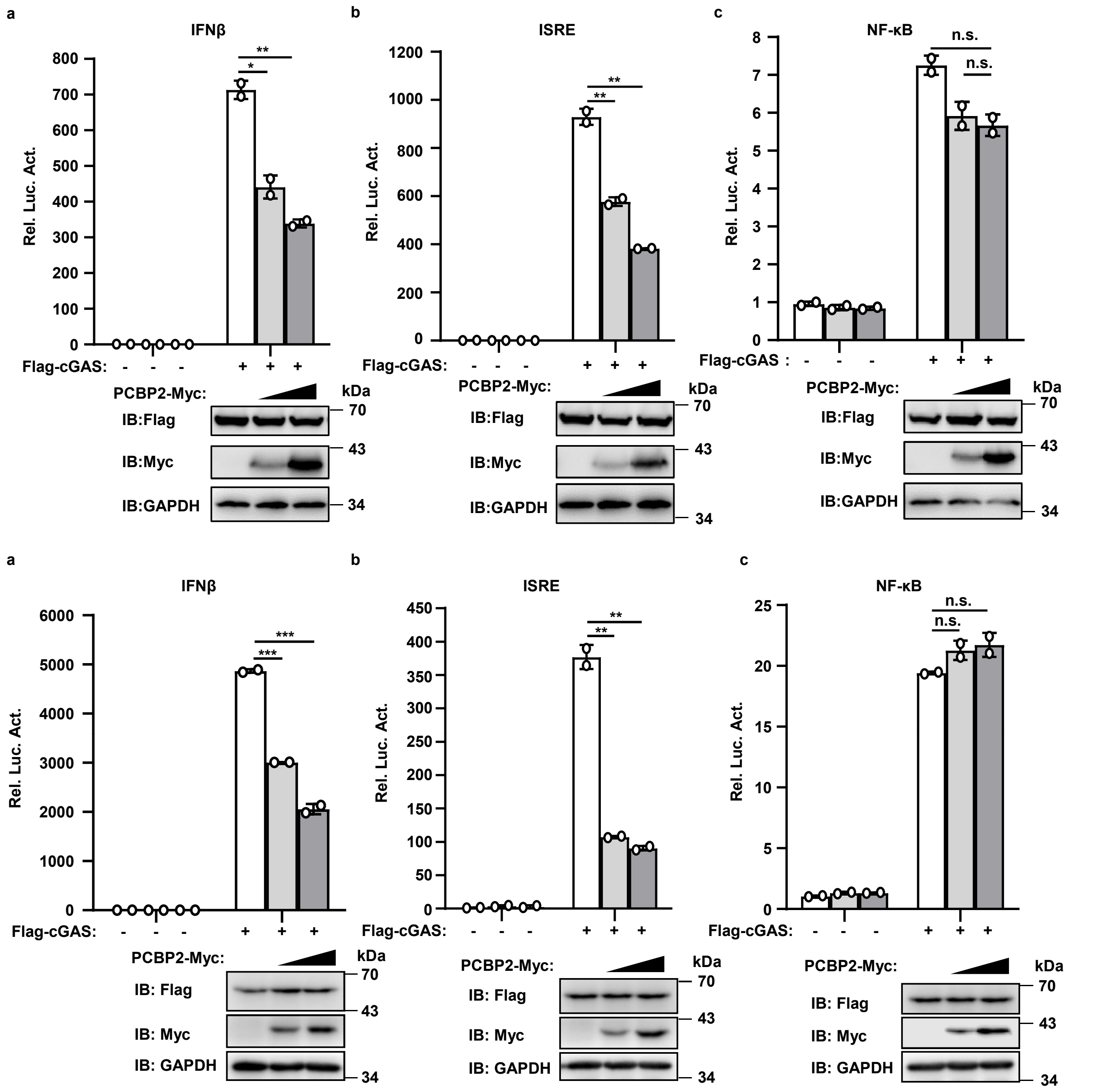

Supplementary Figure 12: The repeated results of Figure 2 a-c.
HEK293T cells stably expressing Flag-STING were transfected with the indicated expression plasmids together with luciferase reporter constructs driven by the promoter of genes encoding IFNβ (**a**), ISRE (**b**), or NF-κB (**c**). Renilla was used as an internal control. Twenty-four hours after transfection, cells were lysed for luciferase reporter assays and immunoblotting. Repeat2 (upper panels): **a** p=0.0114; p=0.0028. **b** p=0.0057, p=0.0018 in sequence. Repeat3 (lower panels): **a** p=0.0002, p=0.0008 in sequence. **b** p=0.0022, p=0.002 in sequence.
Data are presented as mean values ± SD (n=2 independent samples). Two-tailed Student's t-test was used to analyze statistical significance. *$p < 0.05$, **$p < 0.01$, ***$p < 0.001$, n.s. not significant versus the control groups. Source data are provided as a Source data file.

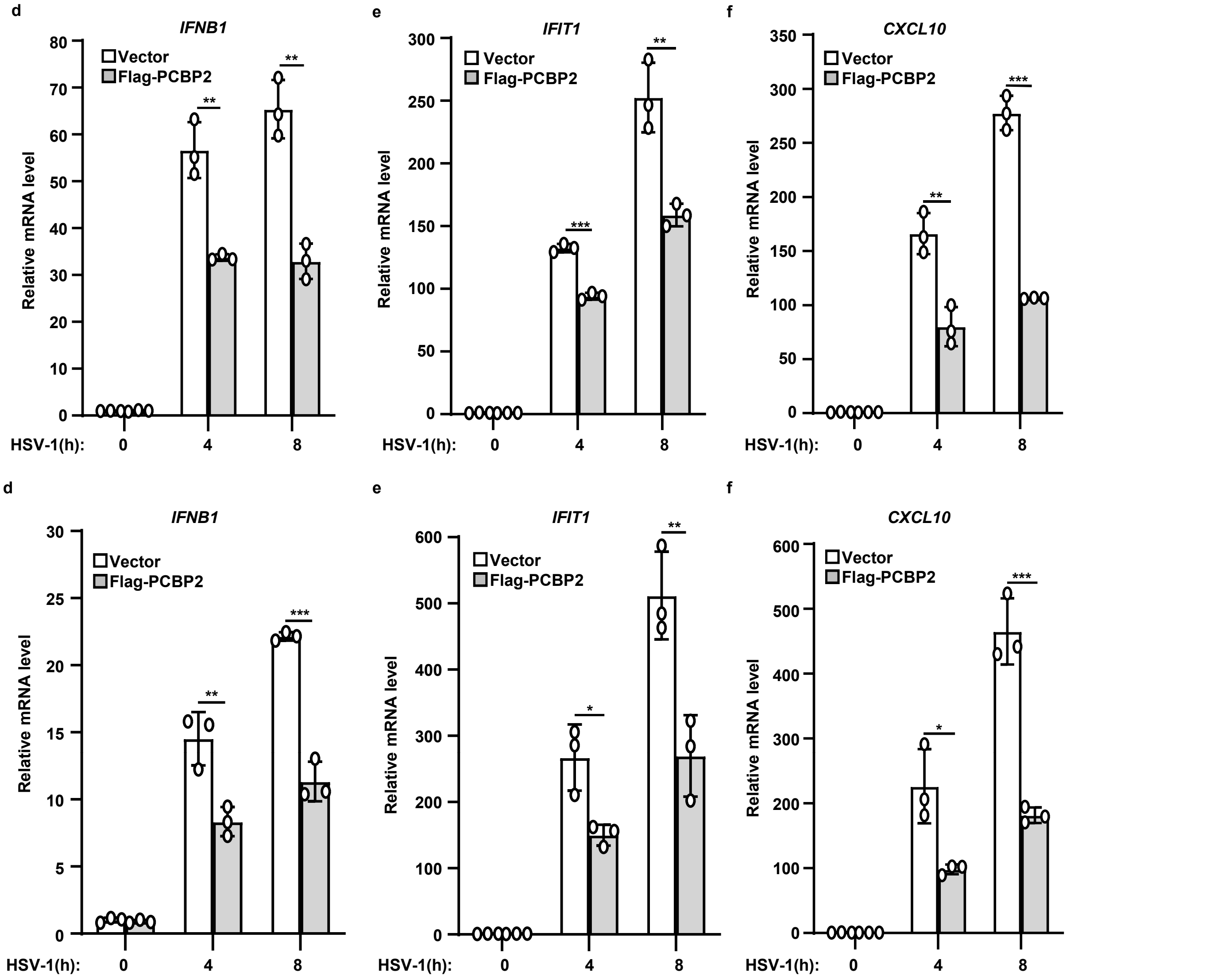

Supplementary Figure 12 continued: The repeated results of Figure 2 d-f.

THP-1 cells were infected with a lentivirus expressing PCBP2 or an empty vector for 48 h, followed by HSV-1 infection (MOI=5) for 10 h. The cells were harvested to isolate RNA, and the transcriptional levels of *IFNB1* (**d**), *IFIT1* (**e**), and *CXCL10* (**f**) were measured by qRT-PCR analysis. Repeat2 (upper panels): **d** p=0.0027 (4h); p=0.0015 (8h). **e** p=0.0001 (4h); p=0.0051 (8h). **f** p=0.0047 (4h); p<0.0001 (8h). Repeat3 (lower panels): **d** p=0.0092 (4h); p=0.0002 (8h). **e** p=0.0179 (4h); p=0.0098 (8h). **f** p=0.0186 (4h); p=0.0007 (8h).

Data are presented as mean values ± SD (n=3 independent samples). Two-tailed Student's t-test was used to analyze statistical significance. *$p < 0.05$, **$p < 0.01$, ***$p < 0.001$. Source data are provided as a Source data file.

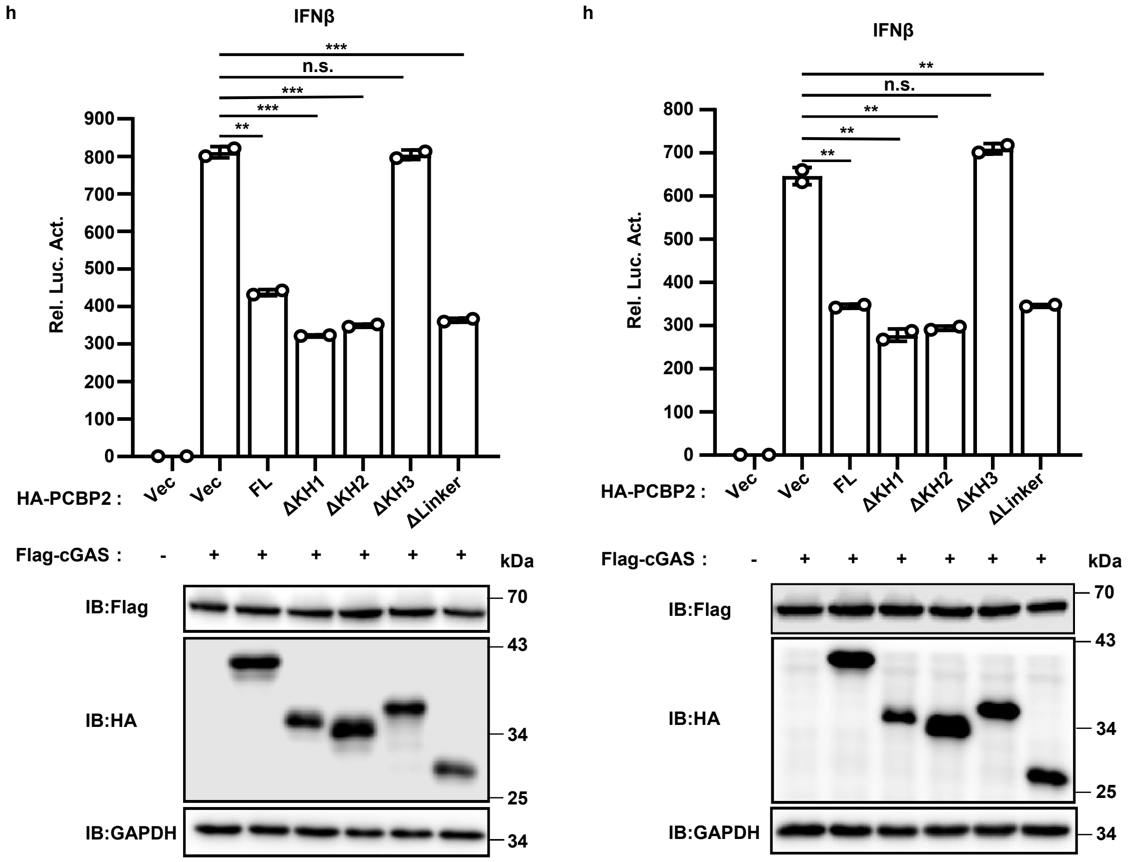

Supplementary Figure 12 continued: The repeated results of Figure 2 h.

HEK293T cells stably expressing Flag-STING were co-transfected with cGAS and PCBP2 or its mutant constructs together with the IFNβ-Luc reporter, followed by immunoblotting with the indicated antibodies. Repeat2 (Left panels): p=0.001 (FL); p=0.0005 (ΔKH1); p=0.0006 (ΔKH2); p=0.6783 (ΔKH3); p=0.0006 (ΔLinker). Repeat3 (Right panels): p=0.0023 (FL); p=0.0022 (ΔKH1); p=0.0017 (ΔKH2); p=0.0621 (ΔKH3); p=0.0023 (ΔLinker).

Data are presented as mean values ± SD (n=2 independent samples). Two-tailed Student's t-test was used to analyze statistical significance. $^{**}p < 0.01$, $^{***}p < 0.001$, n.s. not significant versus the control groups. Source data are provided as a Source data file.

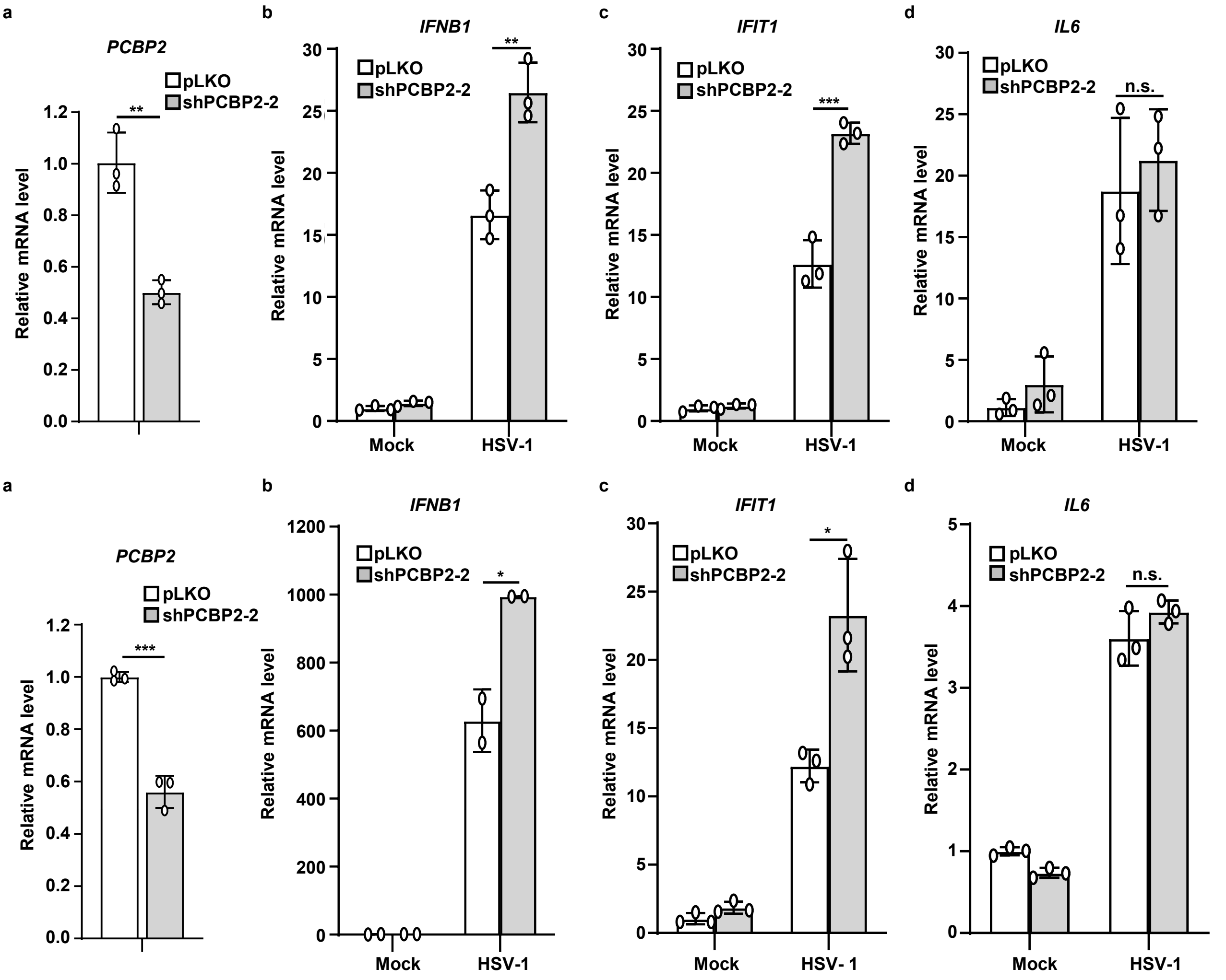

Supplementary Figure 12 continued: The repeated results of Figure 3 a-d.
THP-1 cells were infected with lentiviruses-based shRNA targeting *PCBP2* or an empty vector for 48 h and then left uninfected or infected with HSV-1 (MOI=5) for 6 h. The cells were harvested for qRT-PCR assays to measure the transcriptional levels of *PCBP2* (**a**), *IFNB1* (**b**), *IFIT1* (**c**), and *IL6* (**d**). Repeat2 (upper panels): **a** p=0.0023. **b** p=0.0053. **c** p=0.0009. Repeat3 (lower panels): **a** p=0.0003. **b** p=0.0301. **c** p=0.0112.
Data are presented as mean values ± SD (n=2 or n=3 independent samples). Two-tailed Student's t-test was used to analyze statistical significance. *$p < 0.05$, **$p < 0.01$, ***$p < 0.001$, n.s. not significant versus the control groups. Source data are provided as a Source data file.

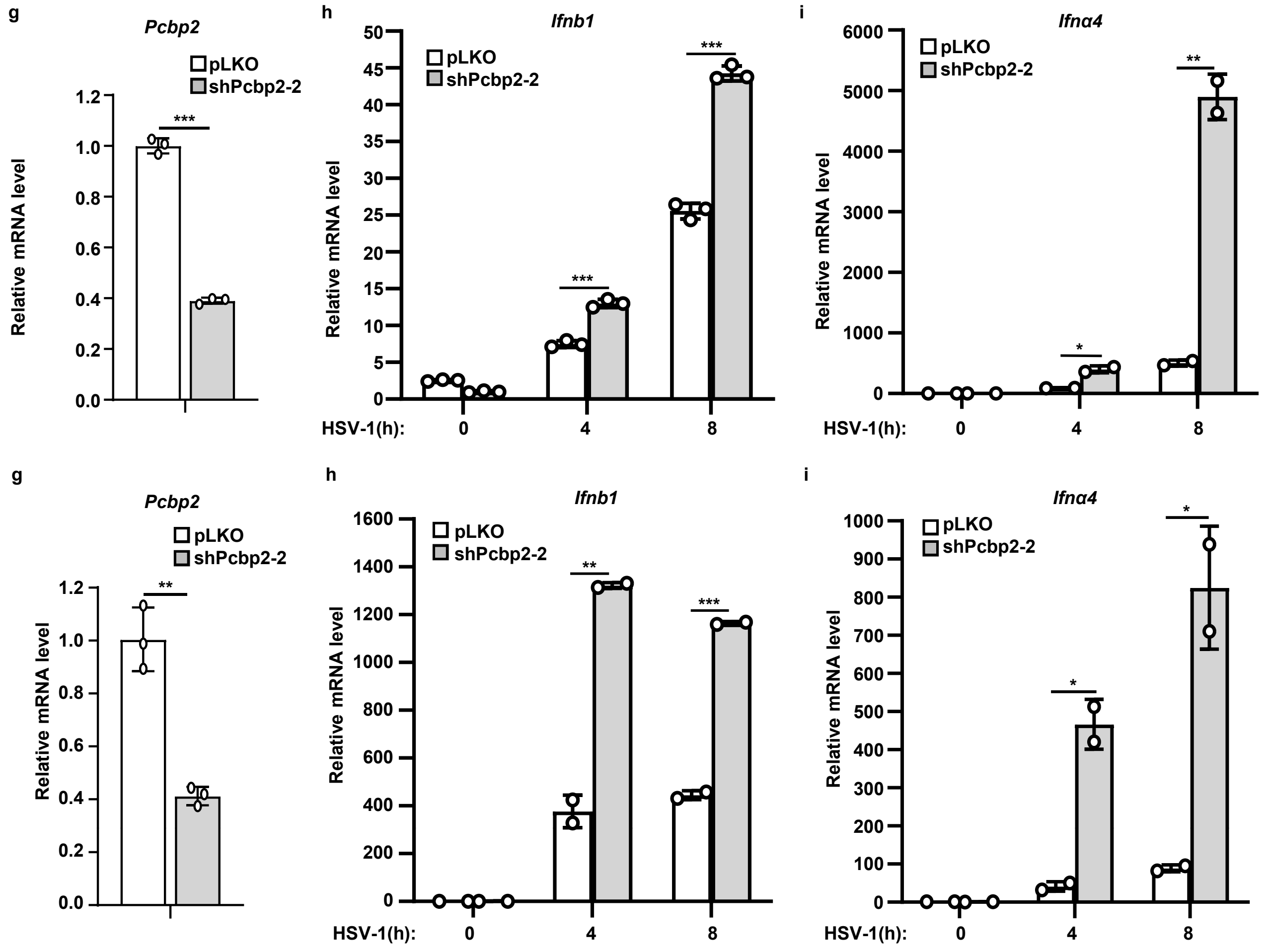

Supplementary Figure 12 continued: The repeated results of Figure 3 g-i.
RAW264.7 cells were infected with a lentivirus-based shRNA targeting *Pcbp2* (sh*Pcbp2*-2) or an empty vector for 48 h and then infected with HSV-1 (MOI=5) for the indicated times. Transcriptional levels of *Pcbp2* (**g**), *Ifnb1* (**h**), and *Ifnα4* (**i**) were detected by qRT-PCR assays. Repeat2 (upper panels): **g** $p<0.0001$. **h** $p=0.0002$ (4h); $p<0.0001$ (8h). **i** $p=0.0165$ (4h); $p=0.0037$ (8h). Repeat3 (lower panels): **g** $p=0.0012$. **h** $p=0.0027$ (4h); $p=0.0004$ (8h). **i** $p=0.0121$ (4h); $p=0.0233$ (8h).
Data are presented as mean values ± SD (n=2 or n=3 independent samples). Two-tailed Student's t-test was used to analyze statistical significance. *$p < 0.05$, **$p < 0.01$, ***$p < 0.001$. Source data are provided as a Source data file.

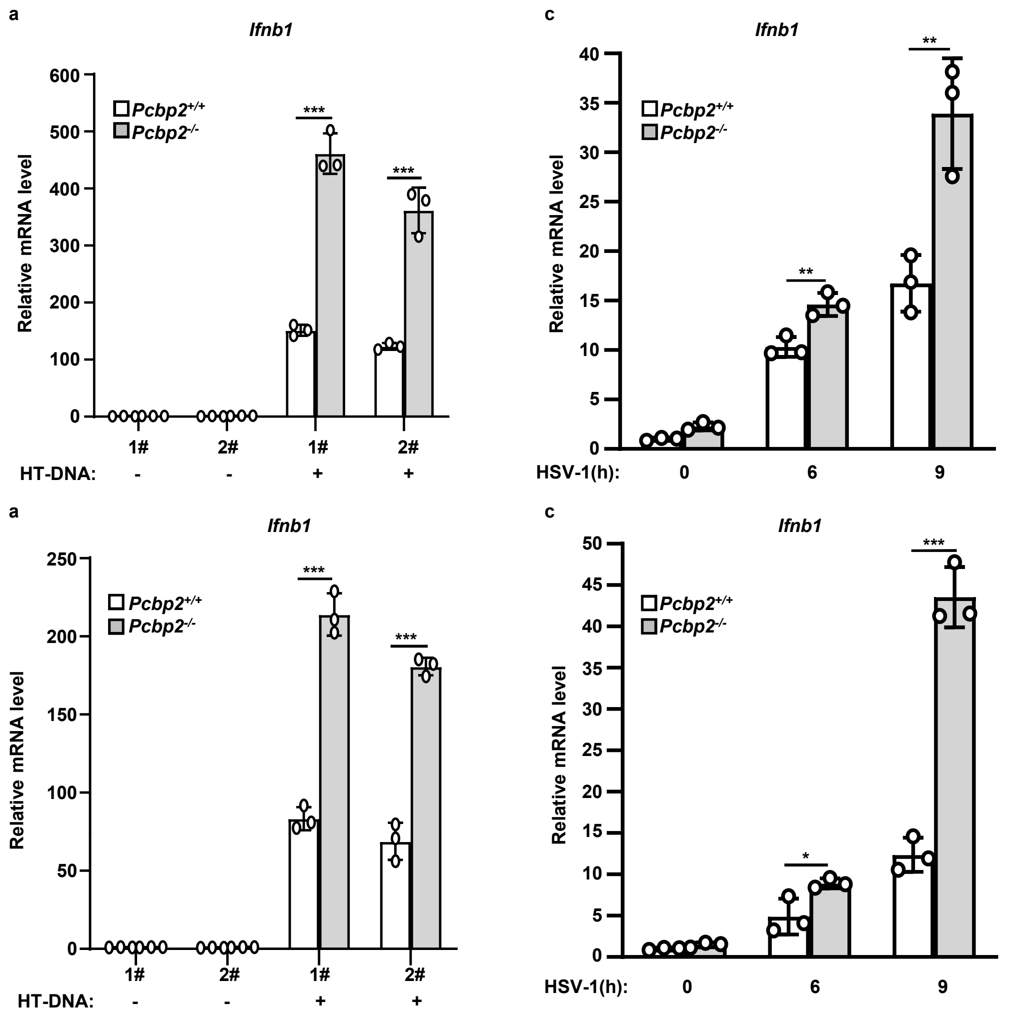

Supplementary Figure 12 continued: The repeated results of Figure 4 a, c.
a Two different clones from wild-type and *Pcbp2*-deficient L929 cells were transfected with HT-DNA (2 μg/ml) for 6 h. The cells were harvested for qRT-PCR analysis to measure the transcriptional level of *Ifnb1*.
c *Pcbp2*$^{+/+}$ and *Pcbp2*$^{-/-}$ L929 cells were infected with HSV-1 (MOI=5) for the indicated times and then harvested for the measurement of *Ifnb1* mRNA levels by qRT-PCR. Repeat2 (upper panels): **a** p<0.0001 (1#); p=0.0005 (2#). **c** p=0.0085 (6h); p=0.0091 (9h). Repeat3 (lower panels): **a** p<0.0001 (1#); p<0.0001 (2#). **c** p=0.0367 (6h); p=0.0002 (9h).
Data are presented as mean values ± SD (n=3 independent samples). Two-tailed Student's t-test was used to analyze statistical significance. *$p < 0.05$, **$p < 0.01$, ***$p < 0.001$. Source data are provided as a Source data file.

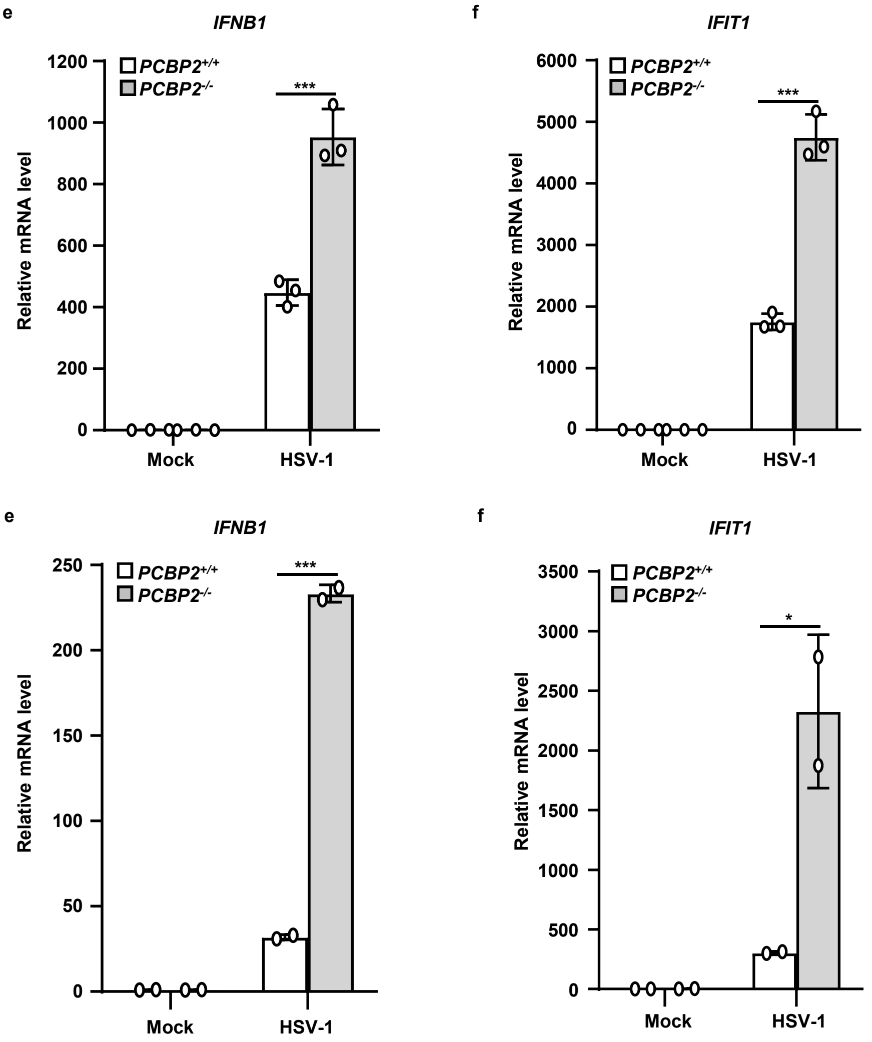

Supplementary Figure 12 continued: The repeated results of Figure 4 e-f.
PCBP2$^{+/+}$ and *PCBP2*$^{-/-}$ THP-1 cells were infected with HSV-1 at an MOI of 5 for 6 h. The cells were harvested for qRT-PCR analysis to measure the mRNA levels of *IFNB1* (**e**) and *IFIT1* (**f**). Repeat2 (upper panels): **e** p=0.0009. **f** p=0.0002. Repeat3 (lower panels): **e** p=0.0003; **f** p=0.0471.
Data are presented as mean values ± SD (n=2 or n=3 independent samples). Two-tailed Student's t-test was used to analyze statistical significance. *$p < 0.05$, ***$p < 0.001$. Source data are provided as a Source data file.

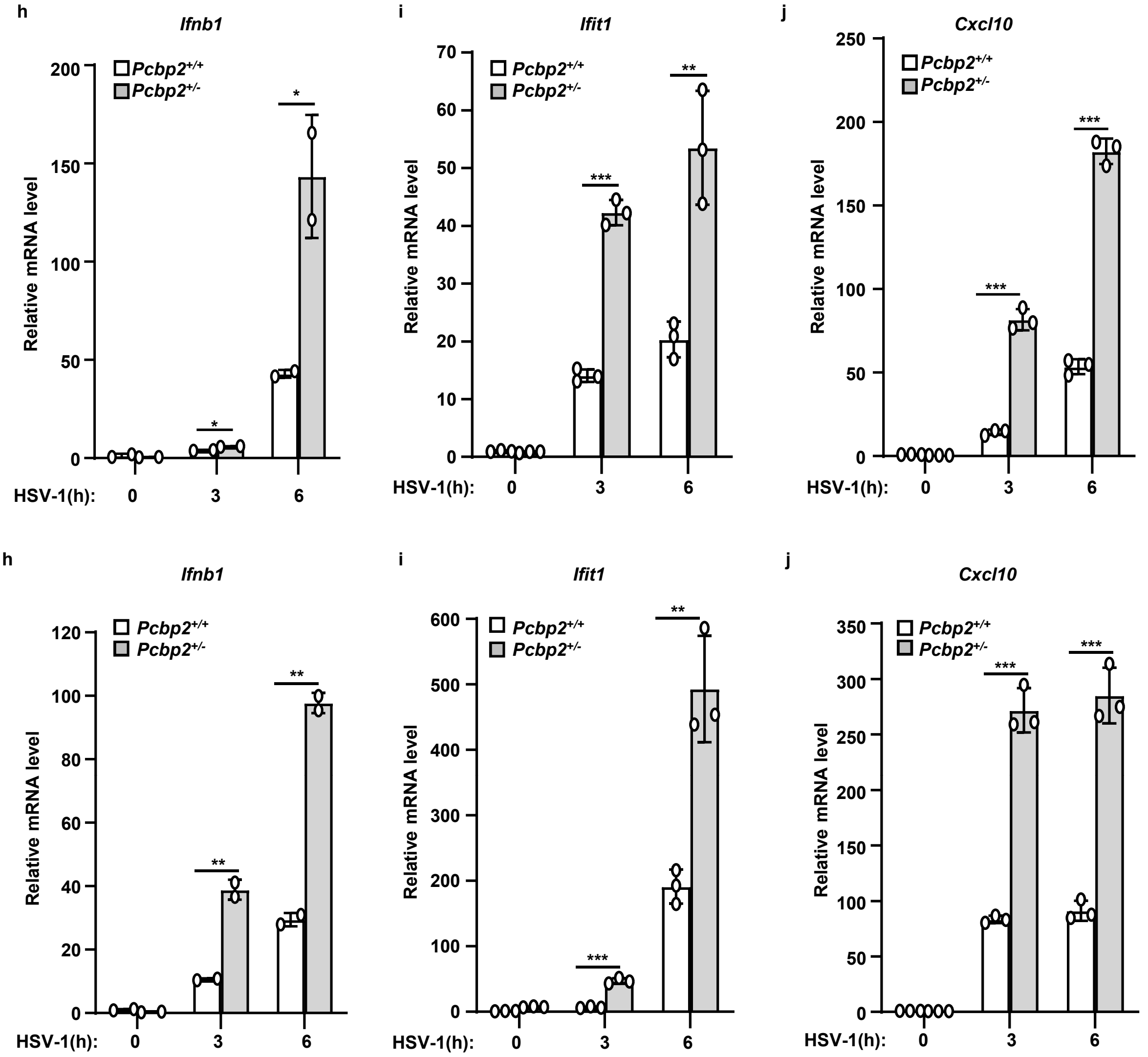

Supplementary Figure 12 continued: The repeated results of Figure 4 h-j.
Pcbp2$^{+/+}$ and *Pcbp2*$^{+/-}$ MEFs were infected with HSV-1 (MOI=5) for the indicated times and then lysed for the quantification of *Ifnb1* (**h**), *Ifit1* (**i**), and *Cxcl10* (**j**) mRNA levels by qRT-PCR. Repeat2 (upper panels): **h** p=0.0112 (3h); p=0.0455 (6h). **i** p<0.0001 (3h); p=0.0051 (6h); **j** p<0.0001 (3h); p<0.0001 (6h). Repeat3 (lower panels): **h** p=0.0063 (3h); p=0.0016 (6h). **i** p<0.0001 (3h); p=0.0036 (6h); **j** p<0.0001 (3h); p=0.0002 (6h).
Data are presented as mean values ± SD (n=2 or n=3 independent samples). Two-tailed Student's t-test was used to analyze statistical significance. *$p < 0.05$, **$p < 0.01$, ***$p < 0.001$. Source data are provided as a Source data file.

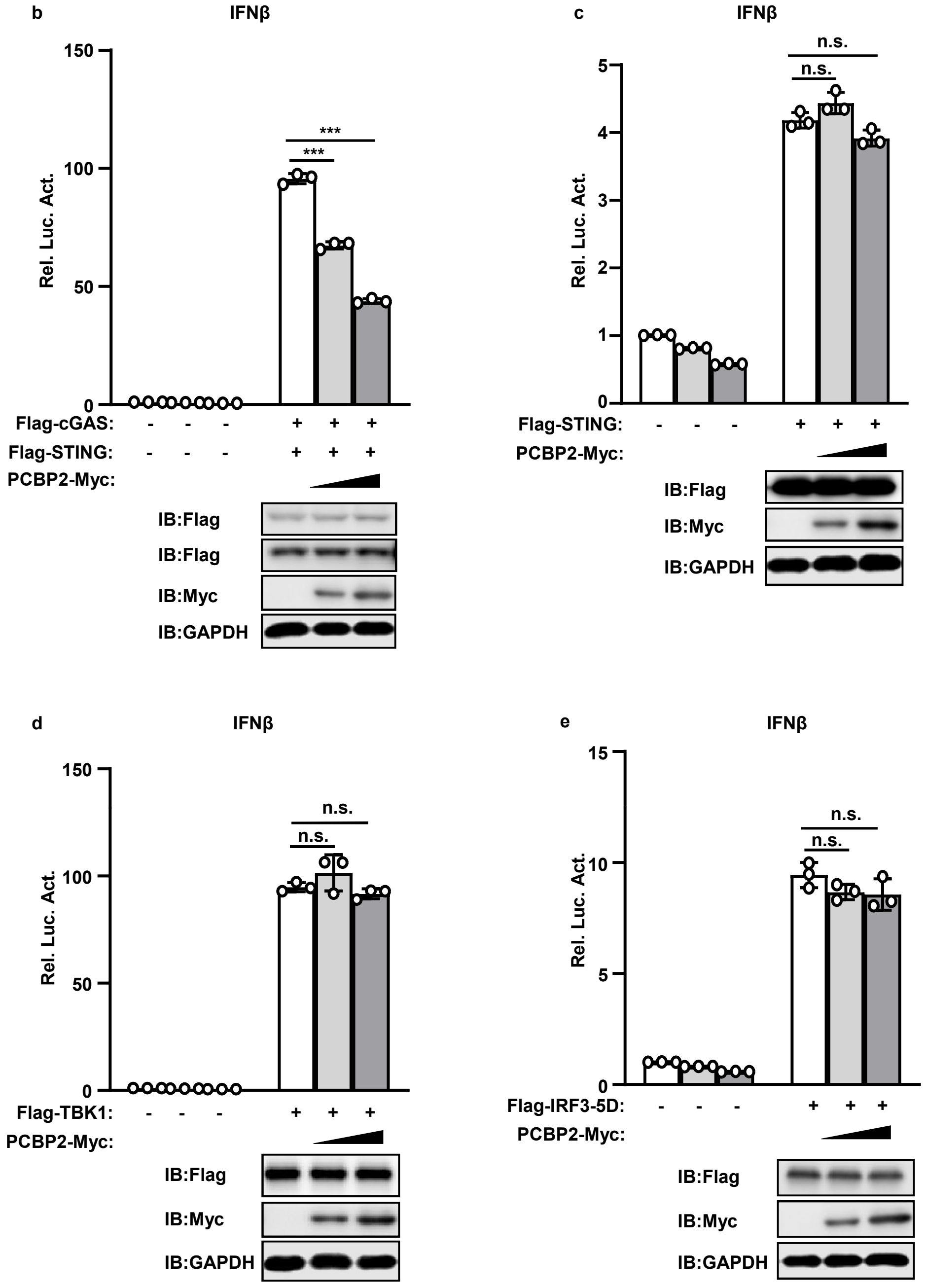

Supplementary Figure 12 continued. The repeated results of Figure 5 b-e

HEK293T cells were transfected with IFNβ-Luc and expression vectors encoding cGAS and STING (**b**), STING (**c**), TBK1 (**d**), or IRF3-5D (**e**), together with the increased amount of PCBP2 or an empty vector. Twenty-four hours after transfection, cells were lysed for luciferase reporter assays (upper panel) and immunoblotting assays (lower panels). **b** $p<0.0001$, $p<0.0001$ in sequence.

Data are presented as mean values ± SD (n=3 independent samples). Two-tailed Student's t-test was used to analyze statistical significance. ***$p < 0.001$, n.s. not significant versus the control groups. Source data are provided as a Source data file.

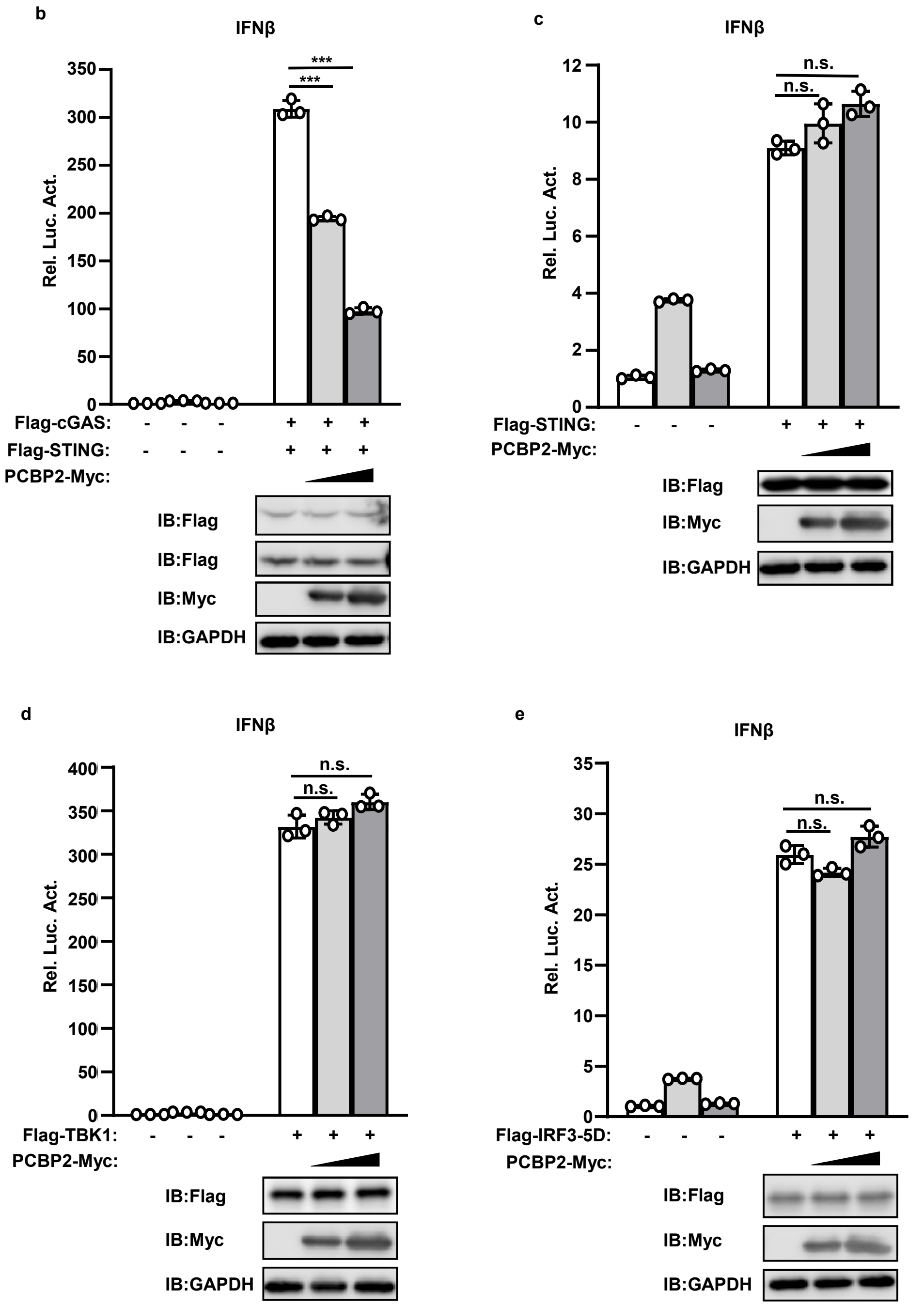

Supplementary Figure 12 continued. The repeated results of Figure 5 b-e
HEK293T cells were transfected with IFNβ-Luc and expression vectors encoding cGAS and STING (**b**), STING (**c**), TBK1 (**d**), or IRF3-5D (**e**), together with the increased amount of PCBP2 or an empty vector. Twenty-four hours after transfection, cells were lysed for luciferase reporter assays (upper panel) and immunoblotting assays (lower panels). **b** $p<0.0001$, $p<0.0001$ in sequence.
Data are presented as mean values ± SD (n=3 independent samples). Two-tailed Student's was used to analyze statistical significance. ***$p < 0.001$, n.s. not significant versus the control groups. Source data are provided as a Source data file.

Supplementary Table 1. Sequences of primers used in qRT-PCR experiments

Gene name	Forward primers	Reverse primer
Human *GAPDH*	ATGACATCAAGAAGGTGGTG	CATACCAGGAAATGAGCTTG
Human *IFNB1*	AGGACAGGATGAACTTTGAC	TGATAGACATTAGCCAGGAG
Human *IFIT1*	GATCTGGAAAGCTTGAGCCT	GGGTGCCTAAGGACCTTG
Human *CXCL10*	GTGGCATTCAAGGAGTACCTC	TGATGGCCTTCGATTCTGGATT
Human *IL6*	AAATTCGGTACATCCTCGACGG	GGAAGGTTCAGGTTGTTTTCTGC
Human *cGAS*	TATAACCCTGGCTTTGGA	GCTTTAGTCGTAGTTGCTTC
Human *PCBP2*	ACTCTCACCATCCGGCTACTT	TGTTGATACGTGCACCACTCT
Mouse *β-Actin*	TCCAGCCTTCCTTCTTGGGT	GCACTGTGTTGGCATAGAGGT
Mouse *Ifnb1*	ATGGTGGTCCGAGCAGAGAT	CCACCACTCATTCTGAGGCA
Mouse *Ifit1*	CTGAGATGTCACTTCACATGGAA	GTGCATCCCCAATGGGTTCT
Mouse *Cxcl10*	GAATCCGGAATCTAAGACCATCAA	GTGCGTGGCTTCACTCCAGT
Mouse *Ifna4*	AGCCTGTGTGATGCAGGAACC	CAGCAAGTTGGTTGAGGAAGAG
Mouse *Il-6*	TCCATCCAGTTGCCTTCTTG	GGTCTGTTGGGAGTGGTATC
Mouse *Pcbp2*	ACTCTCACCATCAGGCTACTT	TGTTGATACGTGCACCACTCT
