# Supplementary material for: PCBP2 maintains antiviral signaling homeostasis by regulating cGAS enzymatic activity via antagonizing its condensation
Source: Nat Commun. 2022 Mar 23;13:1564. doi: 10.1038/s41467-022-29266-9 (PMC8943206; doi:10.1038/s41467-022-29266-9)
Supplement: Supplementary file 10 — Reporting-summary [file 41467_2022_29266_MOESM10_ESM.pdf]

nature portfolio

Corresponding author(s): Qinmiao Sun

Last updated by author(s): 2-8-2022

# Reporting Summary

Nature Portfolio wishes to improve the reproducibility of the work that we publish. This form provides structure for consistency and transparency in reporting. For further information on Nature Portfolio policies, see our Editorial Policies and the Editorial Policy Checklist.

## Statistics

For all statistical analyses, confirm that the following items are present in the figure legend, table legend, main text, or Methods section.

| n/a | Confirmed | |
|---|---|---|
| ☐ | ☒ | The exact sample size (*n*) for each experimental group/condition, given as a discrete number and unit of measurement |
| ☐ | ☒ | A statement on whether measurements were taken from distinct samples or whether the same sample was measured repeatedly |
| ☐ | ☒ | The statistical test(s) used AND whether they are one- or two-sided<br>*Only common tests should be described solely by name; describe more complex techniques in the Methods section.* |
| ☒ | ☐ | A description of all covariates tested |
| ☒ | ☐ | A description of any assumptions or corrections, such as tests of normality and adjustment for multiple comparisons |
| ☐ | ☒ | A full description of the statistical parameters including central tendency (e.g. means) or other basic estimates (e.g. regression coefficient) AND variation (e.g. standard deviation) or associated estimates of uncertainty (e.g. confidence intervals) |
| ☐ | ☒ | For null hypothesis testing, the test statistic (e.g. *F*, *t*, *r*) with confidence intervals, effect sizes, degrees of freedom and *P* value noted<br>*Give P values as exact values whenever suitable.* |
| ☒ | ☐ | For Bayesian analysis, information on the choice of priors and Markov chain Monte Carlo settings |
| ☒ | ☐ | For hierarchical and complex designs, identification of the appropriate level for tests and full reporting of outcomes |
| ☐ | ☒ | Estimates of effect sizes (e.g. Cohen's *d*, Pearson's *r*), indicating how they were calculated |

*Our web collection on statistics for biologists contains articles on many of the points above.*

## Software and code

Policy information about availability of computer code

| | |
|---|---|
| Data collection | Bio-Rad CFX Maestro (BIO-RAD) or Light Cycler 480® (Roche) for qRT-PCR; Tanon 5200 for Western Blot; Zeiss LSM 710 META, ANDOR CR-DFLY-505 and Nikon A1 confocal microscope for confocal; Thermo Proteome Discovery (version 2.3) for mass spectrometry; SAINTexpress (version 3.6.1) software for cGAS-interacting protein Specificity filtering; Cytoscape software (version 3.8.2) for visualization of the cGAS interaction network; Biacore X100 instrument (GE Healthcare) for SPR. |
| Data analysis | Graphpad Prism 8 for statistical analysis; ZEN2012, ImarisViewer 9.5.1 and NIS-Elements AR analysis 5.20.00 64-bit software for confocal image analysis; Biacore X100 evaluation software for SPR; ImageJ (version 1.4.3.67) for immuno-blot and fluorescence intensity quantification; Adobe Premiere Pro 2020 for movie processing. |

For manuscripts utilizing custom algorithms or software that are central to the research but not yet described in published literature, software must be made available to editors and reviewers. We strongly encourage code deposition in a community repository (e.g. GitHub). See the Nature Portfolio guidelines for submitting code & software for further information.

## Data

Policy information about availability of data

All manuscripts must include a data availability statement. This statement should provide the following information, where applicable:

- Accession codes, unique identifiers, or web links for publicly available datasets
- A description of any restrictions on data availability
- For clinical datasets or third party data, please ensure that the statement adheres to our policy

The mass spectrometry proteomics data have been deposited to the ProteomeXchange Consortium via the PRIDE partner repository with the dataset identifier PXD023597 (http://www.ebi.ac.uk/pride/archive/projects/PXD023597). The source data underlying Figures 1a–d, f, g, 2a-h, 3a–i, 4a-l, 5a-j, 6a-f and Supplementary

Figures 2a-c, e, g, 3a–d, 4a-e, 5a-e, 6a-d, 7a, d, e, h, 9a-c, 10a, b, 11a-c and Supplementary Figures 12 are provided as a Source Data file. All unique biological materials used are available from the authors upon reasonable requests.

# Field-specific reporting

Please select the one below that is the best fit for your research. If you are not sure, read the appropriate sections before making your selection.

[x] Life sciences [ ] Behavioural & social sciences [ ] Ecological, evolutionary & environmental sciences

For a reference copy of the document with all sections, see nature.com/documents/nr-reporting-summary-flat.pdf

# Life sciences study design

All studies must disclose on these points even when the disclosure is negative.

| | |
|---|---|
| Sample size | No statistical methods were used to predetermine sample size. Sample size was based on empirical data from previous experimental experience with similar assays. The detail was reported in the Statistical Analysis section. |
| Data exclusions | No data were excluded from the studies |
| Replication | Experiments were repeated at least two times to ensure reproducibility of data as indicated in the Figure Legends. All attempts are successful. |
| Randomization | 12 weeks old male and female PCBP2+/- mice were randomly chosen for breeding to isolate MEFs as described in the text and Methods section. |
| Blinding | Investigators were blinded to mouse chosen. |

# Reporting for specific materials, systems and methods

We require information from authors about some types of materials, experimental systems and methods used in many studies. Here, indicate whether each material, system or method listed is relevant to your study. If you are not sure if a list item applies to your research, read the appropriate section before selecting a response.

## Materials & experimental systems

| n/a | Involved in the study | |
|---|---|---|
| [ ] | [x] | Antibodies |
| [ ] | [x] | Eukaryotic cell lines |
| [x] | [ ] | Palaeontology and archaeology |
| [ ] | [x] | Animals and other organisms |
| [x] | [ ] | Human research participants |
| [x] | [ ] | Clinical data |
| [x] | [ ] | Dual use research of concern |

## Methods

| n/a | Involved in the study | |
|---|---|---|
| [x] | [ ] | ChIP-seq |
| [ ] | [x] | Flow cytometry |
| [x] | [ ] | MRI-based neuroimaging |

## Antibodies

| | |
|---|---|
| Antibodies used | Rabbit anti-phospho-IRF3 (Cat# 4947, 1:1000), rabbit anti-IRF3 (Cat# 4302, 1:1000), rabbit anti-phospho-TBK1 (Cat# 5483, 1:1000) and rabbit anti-cGAS antibodies (Cat# 15102, Cat# 79978, Cat# 31659 (Mouse specific), 1:1000) were from Cell Signaling Technology. Rabbit anti-TBK1 (Cat# ab40676, 1:1000) was from Abcam. Rabbit anti-PCBP2 (Cat# 15070-1-AP, 1:1000) was from Proteintech. Mouse anti-PCBP2 (Cat# sc-101136, 1:1000) was from Santa Cruz Biotechnology. Mouse anti-glyceraldehyde-3-phosphate dehydrogenase (GAPDH) (Cat# KM9002, 1:4000), mouse anti-α-Tubulin (Cat# KM9007, 1:4000) and mouse anti-HA (Cat# KM8004, 1:1000) antibodies were from Sungene Biotechnology. Rabbit anti-Flag (Cat# F7425, 1:1000) antibody was from Sigma. Mouse anti-Flag (Cat# M185-3, 1:1000), rabbit anti-HA (Cat# M132-3, 1:1000), rabbit anti-Myc (Cat# MB2562, 1:1000), mouse anti-Myc (Cat# M192-3, 1:1000) antibodies were from MBL. Mouse anti-V5 (Cat# YM3005, 1:1000) antibody was from ImmunoWay Biotechnology Company. Rabbit anti-PCBP1 (Cat# A1004, 1:1000) antibody was from ABclonal Technology. Mouse/Rabbit anti-cGAS antibody (1:1000 for WB; 1:500 for IP) was prepared in our laboratory, the cGAS antibody was generated by immunizing mice or rabbits with purified human cGAS full-length from E. coli. |
| Validation | The commercial antibodies are well used and reported in lots of previous publications. The validation statement on the manufacturer's website as follows: Rabbit anti-phospho-IRF3 (Cat# 4947; Reactivity: H, M; Application: WB). Rabbit anti-IRF3 (Cat# 4302; Reactivity: H, M, R, Mk; Application: WB, IP). Rabbit anti-phospho-TBK1 (Cat# 5483; Reactivity: H, M; Application: WB, IP, IF, F). Rabbit anti-cGAS antibodies (Cat# 15102; Reactivity: H; Application: WB), (Cat# 79978; Reactivity: H; Application: WB, IHC, IF, F), (Cat# 31659; Reactivity: M; Application: WB, IP). Rabbit anti-TBK1 (Cat# ab40676; Reactivity: M, R, H; Application: IF, WB, IHC). Rabbit anti-PCBP2 (Cat# 15070-1-AP; Reactivity: H; Application: IF, IHC, WB). Mouse anti-PCBP2 (Cat# sc-101136; Reactivity: H, M, R; Application: WB, IP, IF, IHC, ELISA). Mouse anti-GAPDH (Cat# KM9002; Reactivity: H, M, R, Shp; Application: WB). Mouse anti-α-Tubulin (Cat# KM9007; Reactivity: H, Rat, M; Application: WB). Mouse anti-HA (Cat# KM8004; Application: WB, IF). Rabbit anti-Flag |

(Cat# F7425; Application: IF, WB CL, IP, DB). Mouse anti-Flag (Cat# M185-3; Application: WB, IP, FCM, IC, ChIP, Co-IP). Rabbit anti-HA (Cat# M132-3; Application: WB, IP). Rabbit anti-Myc (Cat# 562; Application: WB, IP, IC, IH, ELISA, ChIP). Mouse anti-Myc (Cat# M192-3; Application: WB, IP, FCM, IC, Co-IP, ChIP). Mouse anti-V5 (Cat# YM3005; Application: WB, IP, IF). Rabbit anti-PCBP1 (Cat# A1044; Reactivity: H, M, R; Application: WB, IHC, IF) antibody.

## Eukaryotic cell lines

Policy information about cell lines

| | |
|---|---|
| Cell line source(s) | HEK293T (GNHu17), HeLa (TCHu187), THP-1 (TCHu57), L929 (GNM28), and RAW264.7 (TCM13) cells were obtained from the Shanghai Cell Bank of the Chinese Academy of Sciences (Shanghai, China); HEK293A cells were provided by Dr. Hongyu Deng (Institute of Biophysics, Chinese Academy of Sciences) ( ATCC: CRL-1573), mouse embryonic fibroblasts (MEF) were generated as described in the methods section. |
| Authentication | We didn't authenticate cell lines. |
| Mycoplasma contamination | The cell lines were not tested for mycoplasma. |
| Commonly misidentified lines (See ICLAC register) | No commonly misidentified cell lines were used in this study. |

## Animals and other organisms

Policy information about studies involving animals; ARRIVE guidelines recommended for reporting animal research

| | |
|---|---|
| Laboratory animals | Pcbp2 heterozygous mice were generated via  the CRISPR/Cas9-mediated gene deletion system, homozygous and heterozygous MEF cells were obtained from 13.5-day-old embryos by breeding 12-week-old male and female heterozygous mice. C57BL/6J mice were purchased from the Si Pei Fu Biotechnology. |
| Wild animals | No wild animals were used for this study. |
| Field-collected samples | This study did not involve samples collected from field. |
| Ethics oversight | The protocols for animal studies were approved by the Committee on the Ethics of Animal Experiments of the Institute of Zoology, Chinese Academy of Sciences (Beijing, China) (approval number: IOZ15001). |

Note that full information on the approval of the study protocol must also be provided in the manuscript.

## Flow Cytometry

### Plots

Confirm that:

- [ ] The axis labels state the marker and fluorochrome used (e.g. CD4-FITC).
- [ ] The axis scales are clearly visible. Include numbers along axes only for bottom left plot of group (a 'group' is an analysis of identical markers).
- [ ] All plots are contour plots with outliers or pseudocolor plots.
- [ ] A numerical value for number of cells or percentage (with statistics) is provided.

### Methodology

| | |
|---|---|
| Sample preparation | To sort the mCherry positive Hela cells, cells was digested with trypsin, then washed and resuspended to 10^7 cells/ml with PBS before flow cytometry. |
| Instrument | BD FACSAria™ Fusion |
| Software | BD FACSDiva™ Software |
| Cell population abundance | The positive cell population was determined relative to negative control Hela cell. To ensure the purity of the cell population, positive cell population was sorted twice with an interval of 48h cell culturing. |
| Gating strategy | Forward versus side scatter (FSC vs SSC) gating was used to identify cells of interests and exclude debris or dead cells. A forward scatter area (FSC-A) vs side scatter height (SSC-H) was employed to exclude doublets. For mCherry positive measurement, we used non-transfected cells as negative controls. |

- [ ] Tick this box to confirm that a figure exemplifying the gating strategy is provided in the Supplementary Information.
